# Deciphering gene expression patterns using large-scale transcriptomic data and its applications

**DOI:** 10.1093/bib/bbae590

**Published:** 2024-11-14

**Authors:** Shunjie Chen, Pei Wang, Haiping Guo, Yujie Zhang

**Affiliations:** School of Mathematics and Statistics, Henan University, Jinming Avenue, 475004, Kaifeng, China; School of Mathematics and Statistics, Henan University, Jinming Avenue, 475004, Kaifeng, China; Henan Engineering Research Center for Industrial Internet of Things, Henan University, Mingli Road, 450046, Zhengzhou, China; School of Mathematics and Statistics, Henan University, Jinming Avenue, 475004, Kaifeng, China; School of Mathematics and Statistics, Henan University, Jinming Avenue, 475004, Kaifeng, China

**Keywords:** omics data, gene expression distribution, gene selection, skewness, naïve Bayes, sample classification

## Abstract

Gene expression varies stochastically across genders, racial groups, and health statuses. Deciphering these patterns is crucial for identifying informative genes, classifying samples, and understanding diseases like cancer. This study analyzes 11,252 bulk RNA-seq samples to explore expression patterns of 19,156 genes, including 10,512 cancer tissue samples and 740 normal samples. Additionally, 4,884 single-cell RNA-seq samples are examined. Statistical analysis using 16 probability distributions shows that normal samples display a wider range of distributions compared to cancer samples. Cancer samples tend to favor asymmetric distributions such as generalized extreme value, logarithmic normal, and Gaussian mixture distributions. In contrast, certain genes in normal samples exhibit symmetric distributions. Remarkably, more than 95.5% of genes exhibit non-normal distributions, which challenges traditional assumptions. Furthermore, distributions differ significantly between bulk and single-cell RNA-seq data. Many cancer driver genes exhibit distinct distribution patterns across sample types, suggesting potential for gene selection and classification based on distribution characteristics. A novel skewness-based metric is proposed to quantify distribution variation across datasets, showing genes with significant skewness differences have biological relevance. Finally, an improved naïve Bayes method incorporating gene-specific distributions demonstrates superior performance in simulations over traditional methods. This work enhances understanding of gene expression and its application in omics-based gene selection and sample classification.

## Introduction

Gene expression is a stochastic process that varies over time and across genders, racial groups, and health statuses [1–3]. However, previous investigations have primarily relied on limited snapshot data and assumed normal distributions [4–6]. With advancements in high-throughput sequencing technologies [7, 8], the accumulation of large-scale omics data presents new opportunities to elucidate gene distributions [9], identify informative genes [10–14], classify samples [15, 16], and explore complex diseases such as cancer [17, 18]. Yet, prior studies often focused on specific cancer tissues, adjacent normal tissues, and overlooked racial and gender differences, potentially biased by small sample sizes [19–30]. These challenges underscore the need to decode gene expression distributions and explore their applications through integrated analysis of large-scale omics data.

During the past decades, numerous methods have been developed to explore cancers using omics data. Common approaches include penalized regression models like LASSO [31], elastic net [32], and group LASSO [33], as well as Bayesian algorithms such as naïve Bayes (NB) [34] and its extensions [35, 36]. These methods often assume that gene expression data follow normal distributions [6], which facilitates theoretical analysis and ensures desired statistical properties [4–6]. However, based on the analysis of acute myeloid leukemia, ovarian cancer, and glioblastoma multiforme datasets, Torrente *et al.* [9] found that most genes do not conform to normal distributions. They identified the optimal distribution for each gene as the one yielding the maximum $P$-value from the Kolmogorov–Smirnov (KS) test [37]. However, the KS test merely assesses distribution fit and does not measure goodness of fit. Marko *et al.* [38] further observed that genes in cancer tissues favored complex, heavy-tailed distributions with significant skewness and kurtosis, although their findings were based on limited data [38]. It remains unclear whether these observations hold across large-scale omics datasets. Recently, Zhang *et al.* [39] introduced a statistical method called IDEAS, which summarizes gene expression distributions within individuals and evaluates differences between groups. IDEAS heavily relies on denoising techniques and considers only simple distributions. With the growing availability of omics data, it is both necessary and intriguing to decipher gene expression distributions comprehensively using large-scale omics data.

Motivated by the aforementioned challenges, the emergence of an increasing number of large-scale omics datasets now presents us with unprecedented opportunities to delve deeply into the distributional characteristics of gene expression. Building on these discoveries, this study will leverage large-scale transcriptomic data to conduct a more in-depth analysis of gene expression distributions, aiming to fill gaps in the existing literature and offer new perspectives on understanding the variations in gene expression across different populations and conditions. The contributions of this paper are primarily reflected in the following three aspects:

- Large-scale transcriptomic data are collected, and rigorous procedures are designed to decipher gene distribution patterns. Massive bulk RNA-seq data from TCGA [40] are utilized, categorized by clinical information. For each gene, its distribution is fitted with 16 typical probability distributions (Table 1). In contrast to prior work [9], the Bayesian Information Criterion ($BIC$) is employed to identify the potentially optimal distribution for each gene. Subsequently, the KS test is conducted to statistically evaluate whether the gene adheres to this distribution. The study addresses issues related to sample heterogeneity and elucidate reasons for the paucity of genes following normal distributions. Additionally, by focusing on key genes in breast cancer, the paper also explores differences in gene distribution patterns between bulk RNA-seq and single-cell RNA-seq (scRNA-seq) data.
- A novel metric, termed Skewness Ratio ($SR$), is introduced to quantify the skewness variation of genes between two datasets. This metric focuses on identifying genes that exhibit skewness differences in their expression distributions between two datasets, providing complementary insights to DESeq2 and edgeR [41, 42].
- An Improved Naïve Bayes (INB) classifier that integrates gene-specific distributions is developed. In the INB classifier, the optimal distribution fitted for each gene in a dataset is utilized to estimate the posterior probabilities of samples. Subsequently, samples are classified based on these posterior probabilities. While the INB model maintains the independence assumption among genes and incurs a slightly higher computational cost, it demonstrates superior performance in handling categorical data with diverse distributions. This advantage is particularly pronounced when dealing with non-normally distributed data, leading to more accurate classification results than traditional methods.

**Figure 1:**
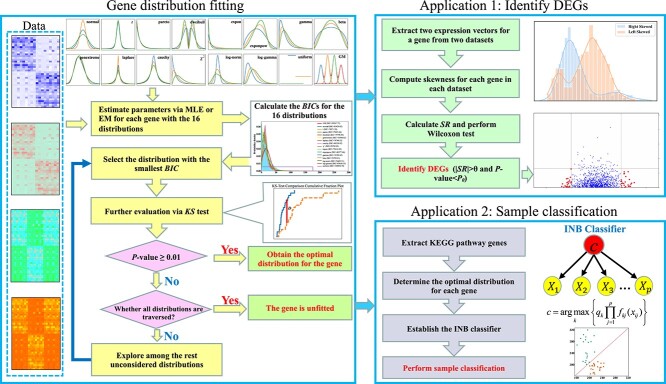
**The schematic diagram illustrates the main contents of this paper.** Based on the collected omics data from various databases, the dataset is segmented into distinct groups based on clinical information. A total of 16 distributions are evaluated to fit gene distributions across these data groups. A rigorous procedure integrating both the BIC and the KS test is employed for distribution selection. Two key applications are subsequently explored. Firstly, leveraging gene distribution variances between two datasets, a novel metric named $SR$ is introduced to identify Differentially Expressed Genes (DEGs) between these datasets. Secondly, an INB classifier, which integrates gene-specific distributions, is proposed for effective sample classification.

The schematic diagram depicting the main contents of this paper is shown in Fig. 1. The key content involves fitting gene-specific distributions, which are then utilized to identify informative genes and classify samples. This approach circumvents the assumption of a universally predefined distribution for all genes in a dataset, particularly challenging the normal distribution assumption. The proposed methods enable in-depth exploration of DEGs and enhance sample classification across diverse datasets.

## Materials and methods

### Data sources

Based on publicly available TCGA [40] database, bulk RNA-seq data for 33 types of cancers are collected, which consists of 11,252 samples, including 10,512 samples for cancer tissues and 740 samples for normal tissues. Gene expression levels were quantified using Transcripts Per Million (TPM) [43]. To focus on reliably expressed genes, those with zero expression in over 95% of the samples are filtered out, resulting in a final set of 19,156 protein-coding genes. The samples can be further stratified based on clinical variables such as gender, health status, race, and cancer stage.

Three publicly available datasets (GSE202203, GSE138536, GSE202695) from the NCBI database are also explored. After filtering out genes with more than 95% zero expression values, 19,644 genes across 3,207 samples from the bulk RNA-seq dataset GSE202203 are considered for breast cancers. GSE138536 and GSE202695 are scRNA-seq datasets. GSE138536 initially includes expression data for 16,408 genes across 1,902 cells, while GSE202695 covers 23,459 genes across 2,982 cells. Genes with zero expression across all samples are excluded, resulting in the consideration of 10,000 genes across 862 cancer cells in GSE138536, and 18,273 genes across 771 cancer cells in GSE202695.

To evaluate the classification performance of the proposed classifier in this study, datasets for multiple cancer types including breast cancer (BRCA), hepatocellular carcinoma (HCC), kidney cancer (KICA), lung adenocarcinoma with lung squamous cell carcinoma (LUAD_LUSC), and thyroid carcinoma with bladder urothelial carcinoma (THCA_BLCA) are examined. BRCA comprises 110 normal samples and 116 cancer samples; HCC includes 50 normal and 50 cancer samples; KICA encompasses 126 normal and 126 cancer samples; LUAD_LUSC consists of 541 samples for lung adenocarcinoma and 502 samples for lung squamous cell carcinoma; THCA_BLCA includes 513 samples for thyroid carcinoma and 412 samples for bladder urothelial carcinoma.

### The Kolmogorov–Smirnov test

The nonparametric KS test [37] assesses whether an observation vector $X_{i}=(x_{1i},x_{2i},\cdots ,x_{ni})^{T}$ for the $i$’th gene is drawn from a distribution $F(x)$. The test statistic is defined as:


(1) \begin{align*}& D=sup_{x}|F_{n}(x)-F(x)|.\end{align*}

Here, $F_{n}(x)$ is the empirical distribution function based on the observations $x_{1i},x_{2i},\cdots ,x_{ni}$, which is defined as:


\begin{align*} &F_n(x)=\frac{1}{n}\sum_{t=1}^nI_{(-\infty,x]}(x_{ti}),\end{align*}

where $I_{(-\infty ,x]}(x_{ti})$ is an indicator function that equals 1 if $x_{ti}<x$, and 0 otherwise. Essentially, $F_{n}(x)$ gives the proportion of observations that are less than or equal to $x$. $F(x)$ is the cumulative distribution function of the theoretical distribution being tested against. The test statistic $D$ (Fig. 1) represents the maximum vertical deviation between the empirical distribution function $F_{n}(x)$ and the theoretical distribution function $F(x)$. It quantifies how well the empirical data matches the theoretical distribution. In this study, if the KS test $P$-value is no less than 0.01, then $X_{i}$ can be considered to follow the distribution $F(x)$.

### Principles for fitting gene expression distributions

Gene expression distributions are fitted using Python’s stats and sklearn.mixture packages [44, 45]. A total of 16 distributions are considered, encompassing normal, $t$, Pareto, double Weibull (dweibull), generalized extreme value (genextreme), Laplace, Cauchy, $\chi ^{2}$, exponential (expon), exponential power (exponpow), gamma, beta, logarithmic normal (log-norm), logarithmic gamma (log-gamma), uniform, and Gaussian mixture (GM) distributions. Table 1 outlines the general forms of their probability density functions (PDFs), covering a broad spectrum of distributions (Supplementary material). During distribution fitting, parameters for GM and dweibull distributions are estimated using the Expectation-Maximization (EM) algorithm, while parameters for the other distributions are estimated via maximum likelihood estimation (MLE).

**Table 1:** General probability density functions (PDFs) of 16 typical distributions. During parameter fitting, certain distributions are simplified and the data undergo preprocessing to conform to these specific distributions. For further details, refer to the Supplementary material.

Distribution	Notation	PDF	Parameter
Normal	$\mathbf{X}\sim N(\mu ,\sigma ^{2})$	$f(x) = \frac{1}{\sqrt{2\pi }\sigma } \exp \left \{-\frac{(x-\mu )^{2}}{2\sigma ^{2}}\right \}$	$\sigma>0,\mu \in R$
$t$	$\mathbf{X}\sim t(m)$	$f(x) = \frac{\Gamma \left (\frac{m+1}{2}\right )}{\sqrt{m\pi } \Gamma \left (\frac{m}{2}\right )}\left (1+\frac{x^{2}}{m}\right )^{-\frac{m+1}{2}}$	$m\in Z^{+}$
Pareto	$\mathbf{X}\sim Pareto(\beta ,A)$	$f(x) =\frac{\beta A^\beta }{x^{\beta +1}}, x\geq A$	$A>0,\beta >0$
Double Weibull	$\mathbf{X}\sim WB(\lambda ,k_{1},k_{2})$	$f(x)=\frac{k_{1}k_{2}}{\lambda }\left (\frac{x}{\lambda }\right )^{k_{1}-1} e^{-(\frac{x}{\lambda })^{k_{1}}}\left [1-e^{-(\frac{x}{\lambda })^{k_{1}}}\right ]^{k_{2}-1}$	$\lambda ,k_{1},k_{2}>0$
General extr. val.	$\mathbf{X}\sim GEV(\mu ,\sigma ,\xi )$	$f(x)=\frac{1}{\sigma }t(x)^{\xi +1}e^{-t(x)},$	$\mu ,\xi \in R, \sigma>0$
		$t(x) =\left (1+\xi \left (\frac{x-\mu }{\sigma }\right )\right )^{-\frac{1}{\xi}},\, \xi \neq 0; t(x) =e^{-\frac{x-\mu}{\sigma}},\, \xi =0.$	
Laplace	$\mathbf{X}\sim Laplace(\mu ,\lambda )$	$f(x)=\frac{1}{2\lambda } \exp \left (-\frac{|x-\mu |}{\lambda }\right )$	$\mu \in R,\lambda>0$
Cauchy	$\mathbf{X}\sim C(\lambda ,\mu )$	$f(x)=\frac{\lambda }{\pi \left (\lambda ^{2}+(x-\mu )^{2}\right )}$	$\lambda>0,\mu \in R$
$\chi ^{2}$	$\mathbf{X}\sim \chi ^{2}(m)$	$f(x) =\frac{1}{2^{m / 2} \Gamma (m / 2)} x^{m / 2-1} \exp (-\frac{x}{2}), x> 0$	$m\in Z^{+}$
Exponential	$\mathbf{X}\sim EXP(\lambda )$	$f(x) =\lambda e^{-\lambda x}, x> 0$	$\lambda>0$
Expon. power	$\mathbf{X}\sim EP(\beta ,\alpha ,\mu )$	$f(x)=\frac{1}{2\alpha \beta ^{1/\beta }\Gamma (1/\beta +1)} \exp \left (-\frac{1}{\beta }|\frac{x-\mu }{\alpha }|^\beta \right )$	$\alpha ,\beta>0,\mu \in R$
Gamma	$\mathbf{X}\sim Gamma(\alpha ,\beta )$	$f(x) =\frac{\beta ^\alpha x^{\alpha -1} exp(-\beta x)}{\Gamma (\alpha )}, x>0$	$\alpha ,\beta>0$
Beta	$\mathbf{X}\sim Beta(\alpha ,\beta )$	$f(x) =\frac{\Gamma (\alpha +\beta ) x^{\alpha -1}(1-x)^{\beta -1}}{\Gamma (\alpha ) \Gamma (\beta )}, 0\leq x\leq 1$	$\alpha ,\beta>0$
Log-norm	$\mathbf{X}\sim LN(\mu ,\sigma ^{2}) $	$f(x) =\frac{1}{\sigma x \sqrt{2 \pi }}\exp \left (-\frac{1}{2}\left (\frac{\ln x-\mu }{ \sigma }\right )^{2}\right ), x>0$	$\sigma>0,\mu \in R$
Log-gamma	$\mathbf{X}\sim L\Gamma (\alpha ,\beta )$	$f(x) =\frac{\beta ^{\alpha }}{\Gamma (\alpha )}(\ln x)^{\alpha -1}\frac{1}{x^{\beta +1}}, x\geq 1$	$\alpha ,\beta>0$
Uniform	$\mathbf{X}\sim U(\alpha ,\beta )$	$f(x) =\frac{1}{\beta -\alpha }, \alpha \leq x\leq \beta$	$\alpha ,\beta \in R$
Gaussian mixture	$\mathbf{X}\sim GM(\mu _{i},\sigma _{i},w_{i},K)$	$f(x) = \sum _{i=1}^{K} w_{i} \frac{1}{\sqrt{2\pi }\sigma _{i}} \exp \left (-\frac{(x-\mu _{i})^{2}}{2\sigma _{i}^{2}}\right )$	$\sigma _{i}>0, w_{i}\geq 0,$
			$\mu _{i}\in R,\sum _{i=1}^{K}w_{i}=1$

Rigorous procedures are developed to determine the optimal distribution for gene $j$ from the 16 distributions (Fig. 1). Initially, the $BIC$ serves as the primary guiding metric:


(2) \begin{align*}& BIC_{j}=-2\ln (L_{j})+k \ln (n).\end{align*}

Here, $L_{j}$ signifies the maximum value of the likelihood function $\Pi _{i=1}^{n}f(x_{ij})$ for the estimated model (Supplementary material), $x_{ij}$ denotes the expression of the $j$’th gene in the $i$’th sample; $k$ denotes the number of parameters in the model, and $n$ is the number of samples. Depending on the distribution considered from Table 1, $f(.)$ and $k$ vary accordingly. Secondly, the distribution with the smallest $BIC$ undergoes further scrutiny with the KS test (Supplementary material). If $P\geq 0.01$ from the KS test, it is designated as the optimal distribution for the gene. Otherwise, among the remaining distributions, the one with the smallest $BIC$ is considered next, if $P\geq 0.01$ from the KS test for this subsequent distribution, it is then defined as optimal. If after evaluating all 16 distributions no $P\geq 0.01$ is obtained from the KS test, then the gene is classified as unfitted (Fig. 1).

### Skewness ratio: an indicator to measure gene distribution differences across datasets

Suppose that $X_{i}^{(l)} (l =D_{1}, D_{2})$ represents the expression sequences of gene $i$ in dataset $l$. The skewness of $X_{i}^{(l)}$ is defined as:


(3) \begin{align*}& Skew(X_{i}^{(l)}) = \frac{1}{n_{i}^{(l)}} \sum_{j=1}^{n_{i}^{(l)}}\left( \frac{x_{ij}^{(l)} - \mu_{i}^{(l)}}{\sigma_{i}^{(l)}} \right)^{3}.\end{align*}

Here, $n_{i}^{(l)}$ represents the number of samples in $X_{i}^{(l)}$, $\mu _{i}^{(l)}$, and $\sigma _{i}^{(l)}$ denote the mean and standard deviation of $X_{i}^{(l)}$, respectively. Based on skewness, $SR_{i}^{(D_{1}D_{2})}$ is defined as follows:


(4) \begin{align*}& SR_{i}^{(D_{1}D_{2})}=\frac{Skew(X_{i}^{(D_{1})})-Skew(X_{i}^{(D_{2})})}{| Skew(X_{i}^{(D_{1})})|+| Skew(X_{i}^{(D_{2})})|}.\end{align*}

$SR_{i}^{(D_{1}D_{2})} \in [-1, 1]$ . $SR_{i}^{(D_{1}D_{2})}> 0 $ indicates that gene $i$ displays stronger skewness within dataset $D_{1}$, while $SR_{i}^{(D_{1}D_{2})} < 0 $ represents a more pronounced skewness in dataset $D_{2}$. Additionally, the Wilcoxon test [46] is performed to validate whether the expression profiles of gene $i$ differ significantly between the two datasets. Based on $SR$ and the Wilcoxon test, genes with $|SR| > 0.5$ and $P$-value$< 0.05$ are defined as DEGs. The threshold values for $|SR|$ and $P$ can be determined based on data analysis and empirical research, analogous to traditional gene differential expression analysis. Higher $|SR|$ and lower $P$ values result in the selection of fewer genes for subsequent analysis, while lower $|SR|$ and higher $P$ values allow for a broader exploration of gene sets.

### The improved naïve Bayes that integrates gene-specific distributions

The NB method is widely employed for sample classification tasks, despite its reliance on several strong assumptions. These assumptions include the independence assumption among different variables and the expectation that all variables adhere to specific distributions [34]. The “naivety” in NB primarily refers to the assumption of conditional independence. Typically, data are assumed to follow normal, multinomial, or Bernoulli distributions when applying the NB method. Despite the challenge of meeting these assumptions in practice, NB remains highly competitive in real-world applications [35].

Statistical learning from large-scale omics data reveals that genes often exhibit diverse distribution patterns, with prevalent distributions including log-norm, genextreme, and GM. Thus, incorporating gene-specific distributions is crucial when applying the NB method. Consequently, an Improved Naïve Bayes (INB) classifier is proposed. This classifier first identifies the appropriate distribution for each gene and subsequently integrates these gene-specific distributions into the classification process. The principles underlying the INB can be summarized as follows: given an observation $X_{(i)}=(x_{i1},x_{i2},\cdots ,x_{ip})^{T}$ for $p$ features, and a class label $c (c=1,2,\cdots ,G)$, based on the NB and conditional probability, the posterior probability classifier can be written as [47]:


(5) \begin{align*}& P(c|X_{(i)})=\frac{q_{c}f_{c}(X_{(i)})}{\sum_{k=1}^{G}q_{k}f_{k}(X_{(i)})}.\end{align*}

Here, $\sum _{k=1}^{G}q_{k}=1$; $q_{k}\in (0,1)$ is the prior probability of class $k$, which can be estimated by historical experience or from training data. $f_{k}(.)$ represents the $p$ dimensional joint PDF for the $k$’th class. Since $\sum _{k=1}^{G}q_{k}f_{k}(X_{(i)})$ serves as a normalizing constant, actually, the posterior probability $P(c|X_{(i)})\propto q_{c}f_{c}(X_{(i)})$. Similar to the traditional NB classifier, the $p$ features in $X_{(i)}$ are also assumed to be mutually independent. Thus, $f_{c}(X_{(i)})$ can be decomposed into the product of marginal PDFs of the $p$ features. That is:


(6) \begin{align*}& f_{c}(X_{(i)}) = \prod_{j=1}^{p} f_{cj}(x_{ij}).\end{align*}

Here, $f_{cj}(.)$ represents the one-dimensional PDF for the $j$’th feature in class $c$. As a result, the posterior probability from the INB can be obtained as:


(7) \begin{align*}& P(c|X_{(i)})=\frac{q_{c}\prod_{j=1}^{p} f_{cj}(x_{ij})}{\sum_{k=1}^{G}q_{k}\prod_{j=1}^{p} f_{kj}(x_{ij})}.\end{align*}

Sample $X_{(i)}$ can be classified into class $c$ if


(8) \begin{align*}& c=\mathop{\arg\max}\limits_{k}\left\{q_{k}\prod_{j=1}^{p}f_{kj}(x_{ij})\right\}.\end{align*}

In real-world applications, the prior probability $q_{k}$ and the PDF $f_{kj}(.)$ can be estimated from the training data. Based on this data, the gene expression distribution for each gene in class $k$ can be estimated, and $f_{kj}(x_{ij})$ can be determined according to the fitted distributions. Given the difficulty in accurately determining cancer incidence rates, the prior probability $q_{k}$ is typically set equally across different classes $k$. The detailed computational processes of the INB can be found in the Supplementary material, and the workflow of the INB is shown in Algorithm 1.




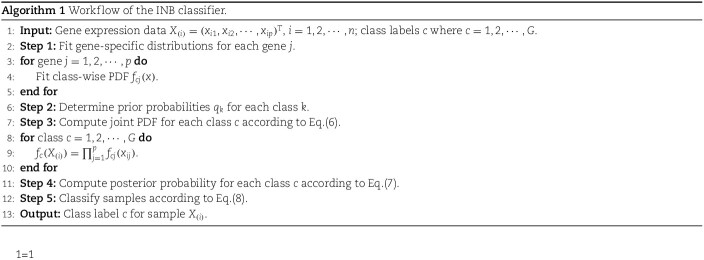



It’s important to note that the INB requires fitting gene distributions, making its computational complexity higher than that of the traditional NB, especially when dealing with a large number of genes. To mitigate this complexity, only genes involved in KEGG pathways associated with cancers are considered. By leveraging the fitted distributions of pathway genes and observed data from a new sample, the posterior probability that the sample belongs to the associated cancer class can be computed.

### Protein–protein interaction network and gene co-expression network

The protein–protein interaction network is curated from the STRING database [48], including only interaction pairs with a confidence score of 0.15 or higher. Gene co-expression relationships are predicted using partial KCC (Supplementary material), with consideration given to gene pairs having an absolute partial KCC greater than 0.1.

### Other statistical analysis

The Pearson’s $\chi ^{2}$ test [49] is performed when evaluating the independence between rows and columns for a contingency table of the 16 distributions in two datasets. The Wilcoxon rank-sum test [50] is performed to verify whether the expression vectors of a gene in two datasets have significant differences. KEGG and GO enrichment analyses are conducted using the clusterProfiler package [51] in R, employing the hypergeometric test.

## Results

### Deciphering gene expression distributions using large-scale transcriptomic data

#### Distributions of cancer driver genes exhibit notable differences between cancerous and normal tissues

**Figure 2:**
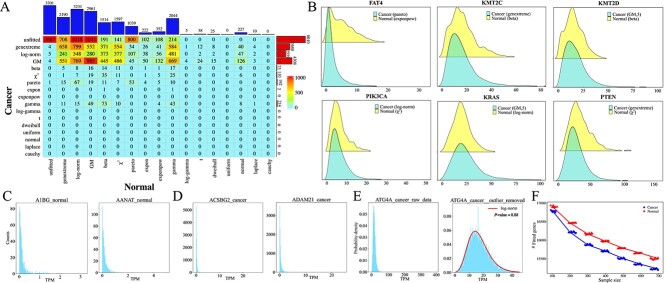
**Comparing the fitted distributions between cancer and normal datasets. A**. Contingency table summarizing the fitted gene distributions in both datasets. **B**. The fitted distributions of cancer driver genes FAT4, KMT2C, KMT2D, PIK3CA, KRAS, and PTEN in the two datasets. $P$-values from Wilcoxon test of the fitted distributions from the two datasets for these genes can be seen in **Supplementary Table S1**. Similarly hereinafter. **C**. The histograms of the unfitted genes A1BG and AANAT in normal samples. **D**. The histograms of the unfitted genes ACSBG2 and ADAM21 in cancer samples. **E**. The unfitted gene ATG4A in cancer samples can be fitted with a log-norm distribution after replacing outliers with its median expression values. **F**. The number of fitted genes decreases with increasing sample sizes, considering randomly sampled data from the two datasets under different sample sizes.

Based on the proposed principles for fitting gene distributions (**Materials and Methods**), distributions of genes in cancer and normal tissues are fitted with 16 typical distributions (including normal, $t$, Pareto, dweibull, genextreme, Laplace, Cauchy, $\chi ^{2}$, expon, exponpow, gamma, beta, log-norm, log-gamma, uniform, and GM; see Table 1). The contingency table illustrating distributions for the 19,156 genes in the two datasets is presented in Fig. 2A. The ratios of genes in cancer samples that follow the 16 distributions are similar to those in normal samples (KS test, $P=0.1061$). An independence test between the rows and columns of the contingency table indicates that the distributions of genes in cancer samples are correlated with those in normal samples ($\chi ^{2}$ test, $P<0.01$). Fitting results for 5,999 (including 3,687 unfitted genes in both datasets) out of the 19,156 genes are consistent across the two datasets. However, 19.32% and 41.81% of genes in normal and cancer samples, respectively, cannot be fitted with any of the 16 distributions. Compared to cancer samples, noticeably more genes in normal samples are fitted with a distribution. Specifically, a considerable number of genes originally following log-norm, GM, beta, $\chi ^{2}$, gamma, Pareto, normal, exponpow, expon distributions in normal samples are transformed into genextreme, log-norm, GM distributions or cannot be fitted with any of the 16 distributions in cancer samples. This underscores the alteration of gene expression patterns from normal to cancerous states.

Interestingly, the distributions of several known cancer driver genes exhibit significant differences between cancer and normal tissues, such as FAT4, KMT2C, KMT2D, KRAS, PIK3CA, and PTEN [52] (Fig. 2B and **Supplementary Table S1**). It is found that FAT4 follows a Pareto distribution (KS test, $P=0.0136$) in cancer samples, while it follows exponpow distribution (KS test, $P=0.8256$) in normal samples. KMT2C exhibits a genextreme distribution (KS test, $P=0.0696$) and a beta distribution (KS test, $P=0.2349$) in cancer and normal samples, respectively. Previous studies indicate that mutations in FAT4 may contribute to the development of 24 types of cancers, while mutations in KMT2C may lead to 32 types of cancers [52].

Analysis of unfitted genes reveals that most of them are centered at low expression values and exhibit heavy-tailed distributions influenced by outliers (Fig. 2C, D). The distribution patterns of these unfitted genes indicate that outliers play a significant role in determining whether they can be fitted with one of the 16 distributions. For example, in cancer samples, ATG4A cannot be fitted with any distribution, and the variance of its expression profiles is 87.70. However, after removing outliers (by z-scoring expression levels and replacing absolute scores greater than 3 with the median expression value), its variance reduces to 55.07. Subsequent fitting analysis suggests that a log-normal distribution is optimal (Fig. 2E). This suggests that outliers may indeed contribute to genes being unfitted.

To investigate whether sample sizes affect the optimally fitted distributions, two experiments are conducted. Firstly, 740 samples are randomly selected 20 times from the 10,512 cancer samples. The results show that on average, 20.78% of genes with unknown distributions are observed, which is significantly lower than the proportion observed in all cancer samples (8010/19156$\approx $41.81%). Secondly, an analysis of the evolution of the number of fitted genes indicates a roughly linear decrease with sample sizes (Fig. 2F). These findings suggest that sample sizes may indeed impact the fitting of gene distributions.

#### Gender-based differences in gene distributions

There are notable gender differences in the incidence and mortality rates of cancer [2, 3, 19, 21–23, 53]. In certain cancers such as bladder, colorectal, and liver cancers, the incidence and mortality rates are higher in males compared to females [2, 3]. These differences may be influenced by hormone levels, gene expression on sex chromosomes, or lifestyle variations among the populations [21–23].

**Figure 3:**
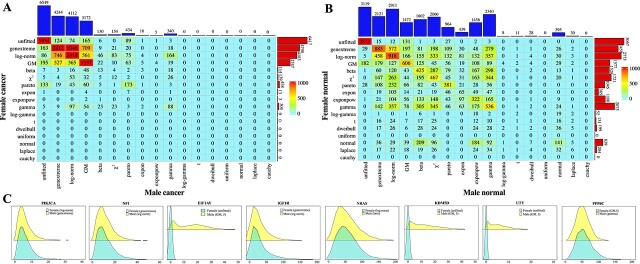
**Disparities in fitted distributions between female and male samples. A**. Gene distribution contingency table in female and male cancer samples. **B**. Gene distribution contingency table in female and male normal samples. **C**. The distributions of genes PIK3CA, NF1, EIF1AY, IGF1R, NRAS, KDM5D, UTY, and PPP6C exhibit gender differences in cancer samples between males and females.

In this section, variations in gene distribution patterns among 4,969 male cancer samples, 5,307 female cancer samples, 370 female normal samples, and 352 male normal samples are explored. Genes in male and female cancer samples primarily exhibit log-normal, genextreme, and GM distributions. Figures 3A and B depict contingency tables of gene distributions for male cancer versus female cancer samples, and male normal versus female normal samples. Independence test between the rows and columns of the contingency tables indicates that the distributions of genes in male samples are correlated with those in female samples ($\chi ^{2}$ test, $P\!<\!2.2e-16$ for both cancer and normal samples). Between the two cancer datasets, only 7,053 genes exhibit identical distributions, while 63.18% of genes show different distributions or are unfitted. Similarly, in male and female normal samples, only 4,755 genes have identical distributions, with 75.18% of genes exhibiting different distributions or being unfitted. The contingency tables for cancer and normal samples differ, with more enriched distributions observed in normal samples. Notably, the numbers of genes following exponpow, gamma, beta, $\chi ^{2}$, expon, normal, and Laplace distributions are notably higher in normal samples compared to cancer samples.

Interestingly, while similar distribution patterns are observed in both male and female cancer samples, as well as in their respective normal samples (Fig. 3A and B), certain differences are notable. For instance, 839 genes are optimally fitted with normal distributions in female normal samples, whereas only 395 genes exhibit normal distributions in male normal samples. Among the 839 genes following normal distributions in female normal samples, 39, 184, 209, 96, 92, 36, 29, 1, 7, and 5 genes are transformed into GM, exponpow, beta, $\chi ^{2}$, gamma, genextreme, log-norm, log-gamma, dweibull, and Laplace distributions, respectively, in male normal samples (Fig. 3B). Only 141 genes retain normal distributions in male normal samples. Moreover, several confirmed gender-related carcinogenic genes exhibit completely different distributions in male and female cancer samples, such as PIK3CA, NF1, EIF1AY, IGF1R, NRAS, KDM5D, UTY, and PPP6C (Fig. 3C). In male and female cancer samples, PIK3CA follows a genextreme distribution (KS test, $P= 0.4623$) and a log-norm distribution (KS test, $P= 0.2202$), respectively. Previous studies have reported somatic mutations and amplification of the PIK3CA gene in various human cancers [54–56], including breast cancer, which is particularly prevalent among women. It has also been noted that amplification of the PIK3CA locus 3q25 is more frequent in female kidney renal clear cell carcinoma patients compared to male patients [19]. Another gene, NF1, exhibits a log-norm distribution (KS test, $P= 0.4167$) in male cancer samples and a genextreme distribution (KS test, $P= 0.0470$) in female cancer samples. NF1 encodes neurofibromin 1, a GTPase-activating protein and a key negative regulator of the RAS and PI3K signaling pathways [57]. Moreover, genes KDM5D, UTY, and NF1 have demonstrated gender differences in lung cancer [21], while PPP6C and IGF1R exhibit sex-biased expression in melanoma [22], and the NRAS gene may play sex-specific roles in acute myelogenous leukemia [23].

In summary, analyses of gene distributions in large-scale omics data reveal that PIK3CA, NF1, EIF1AY, IGF1R, NRAS, KDM5D, UTY, and PPP6C display gender-specific expression patterns, indicating their potential crucial roles in gender-related cancers.

#### Gene distributions exhibit racial disparities

**Figure 4:**
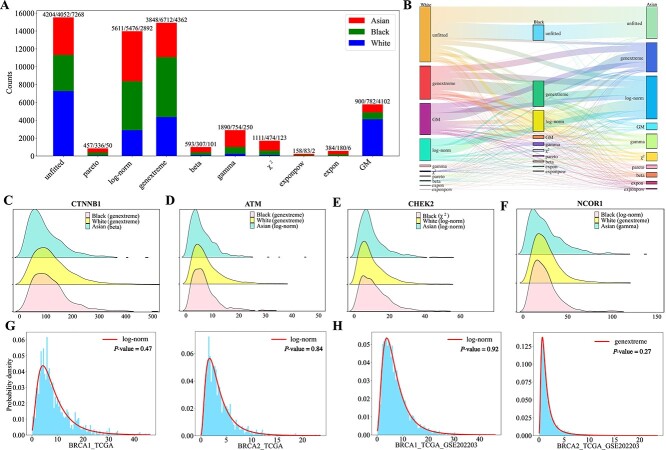
**Racial disparities of gene distributions, and distribution analysis of BRCA1 and BRCA2 in breast cancer datasets. A**. The numbers of genes with fitted distributions in black, white and Asian cancer datasets. **B**. Sankey plots illustrating the distributions of genes among the three racial groups. **C**–**F**. Fitted distributions of genes CTNNB1, ATM, CHEK2, and NCOR1 in the three datasets. **G**. Fitted distributions of BRCA1 and BRCA2 in the breast cancer dataset. **H**. Fitted distributions of BRCA1 and BRCA2 by merging the TCGA data and the GSE202203 breast cancer dataset.

Gene distributions across different racial groups are investigated using a total of 9,340 cancer samples with varying ethnicities, including 7,758 samples from white individuals, 927 samples from black individuals, and 655 samples from Asians. It is observed that in samples from white, black, and Asian individuals, 15.10%, 28.59%, and 29.29% of genes follow log-norm distributions, respectively, while 22.77%, 35.04%, and 20.09% of genes follow genextreme distributions (Fig. 4A). Among the three racial groups, 2,535 genes exhibit identical distributions, whereas the distributions of 4,558 genes are specific to particular racial groups. The most prevalent invariant distributions across the three racial groups are genextreme and log-norm distributions, with a significant number of genes that remained unfitted in the white group also being unfitted in the other two groups (Fig. 4B).

The distribution patterns of several known cancer driver genes, including CTNNB1, ATM, CHEK2, and NCOR1, exhibit racial disparities across datasets (Fig. 4C–F). Gene CTNNB1 follows genextreme distributions in both white and black cancer samples (KS test, $P=0.1045$ and $0.7404$), while it follows a beta distribution in Asian cancer samples (KS test, $P=0.5109$). Studies on endometrial cancer have shown that CTNNB1 mutations are more frequent in Asian individuals compared to black and white individuals [28]. Gene ATM follows a genextreme distribution in both white and black cancer samples (KS test, $P=0.6566$ and $0.2219$, respectively), whereas in Asian cancer samples, it follows a log-norm distribution (KS test, $P=0.7049$). Investigations on bladder cancer have revealed a significantly higher incidence of ATM mutations and associated somatic copy number alterations in white and black populations compared to Asians [26]. Similarly, studies on prostate cancer have reported a higher frequency of ATM gene mutations in white and black populations compared to Asians [27]. Gene CHEK2 follows a $\chi ^{2}$ (KS test, $P=0.8578$), log-norm (KS test, $P=0.1129$), and another log-norm distribution (KS test, $P=0.6103$) in black, white, and Asian cancer samples, respectively. Research on breast cancer has indicated that pathogenic variants of CHEK2 are significantly less frequent in blacks compared to whites [25]. The distribution of the NCOR1 gene in white cancer samples follows a genextreme distribution (KS test, $P=0.1015$), while in Asian cancer samples it follows a gamma distribution (KS test, $P=0.8855$), and in black cancer samples it follows a log-normal distribution (KS test, $P=0.7598$). Previous studies have reported a significantly higher mutation frequency of NCOR1 in triple-negative breast cancer cases among white populations compared to black populations [24].

In summary, gene distributions reveal racial disparities, with several cancer driver genes exhibiting different distributions across racial groups. These findings suggest that these genes may contribute to variations in cancer incidence among different ethnic groups [58].

#### Exploring gene distribution patterns across datasets

This subsection further explores two questions: 1) Whether the distributions of cancer driver genes vary with the sources of samples? 2) Whether gene distribution patterns in bulk RNA-seq and scRNA-seq datasets are consistent?

For the first question, a total of 591 canonical cancer driver genes from the NCG database [59] are investigated in both cancer and normal samples. Our analysis reveal that only 120 genes exhibited similar distributions in both datasets, while the remaining 471 genes are fitted with distinct distributions. When comparing distributions between male and female cancer samples, 364 out of the 591 canonical cancer drivers shared the same distribution patterns. This suggests significant distributional differences for a substantial number of canonical cancer driver genes across datasets, which can aid in sample classification. Using the well-known breast cancer suppressor genes BRCA1 and BRCA2 [60, 61] as examples, in 1,118 breast cancer samples, both genes are fitted with log-norm distributions (Fig. 4G). However, when incorporating the bulk RNA-seq dataset GSE202203 with an additional 3,207 breast cancer samples [62], the distribution fitting reveals that BRCA1 still follows a log-normal distribution, while BRCA2 shifts to a genextreme distribution (Fig. 4H). These findings indicate that the sources of samples may influence the fitted distributions of cancer driver genes.

For the second question, two additional scRNA-seq datasets, GSE138536 and GSE202695 [63, 64], are examined. In GSE138536, only 83 out of 10,000 genes can be fitted with one of the 16 distributions, while in GSE202695, only 541 out of 18,273 genes can be fitted with an optimal distribution. The majority of genes in these datasets cannot be fitted with one of the 16 distributions, indicating that the distribution patterns of most genes in scRNA-seq datasets differ from those in bulk RNA-seq datasets.

In conclusion, gene distribution patterns vary significantly across datasets, providing valuable insights for distinguishing between different datasets.

#### Diversity of gene expression distributions across different developmental stages of cancers

Heterogeneity of gene expression patterns can be influenced by the developmental stages of cancers [65–67]. To explore this heterogeneity, 4,913 out of the 7,758 white cancer samples with developmental stage information are analyzed. The samples are divided into eight subgroups based on gender and cancer stage (Table 2). Each subgroup represents a relatively homogeneous dataset.

**Table 2:** The number of genes following each distribution at different developmental stages of cancer for female and male white cancer patients.

Dataset	#samp.	unfit.	$t$	pareto	log-norm	genextr.	dweibull	beta	log-gamma	gamma	$\chi ^{2}$	lapl.	norm.	exponpow	expon	GM
Fem. stage I	727	3662	107	1093	4526	6541	41	332	0	980	670	22	9	90	175	908
Fem. stage II	829	3794	91	1232	4350	6458	31	383	1	998	577	17	7	60	198	959
Fem. stage III	600	3696	25	1389	4717	5444	3	406	0	1369	920	2	5	143	286	751
Fem. stage IV	212	2953	29	1256	4029	4945	17	582	0	1738	1515	20	62	782	501	727
Male stage I	762	3843	7	1361	4889	5233	1	383	0	1378	782	1	0	71	264	943
Male stage II	675	3874	12	1488	4209	6629	0	396	0	865	544	0	1	107	322	709
Male stage III	679	3874	8	1462	4784	5700	1	386	0	1126	734	0	0	118	316	647
Male stage IV	429	3439	14	1231	4684	6471	4	353	0	1089	704	7	3	196	328	633

**Figure 5:**
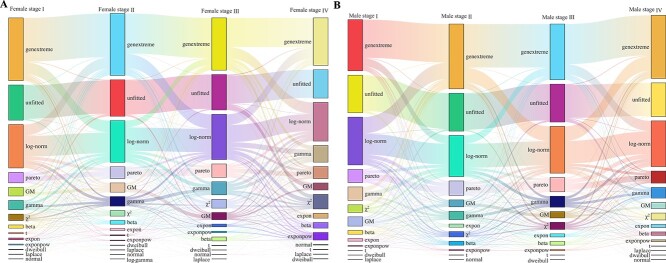
**Sankey plots for the distributions of genes at different developmental stages of cancers. A**. Cases for female cancer samples. **B**. Cases for male cancer samples.

Table 2 presents the number of genes fitted with each distribution across the eight datasets. Notably, compared to the results observed in various cancer datasets in the above subsections, a significant increase in the number of genes (approximately 15,000 out of 19,156 genes) exhibiting fitted distributions is observed. These findings suggest that sample heterogeneity may influence gene distribution patterns. Sankey plots illustrating the distributions of genes at different developmental stages of cancers in female and male cancer samples are depicted in Fig. 5. The figure reveals that many genes follow genextreme and log-norm distributions across different cancer developmental stages, with transitions observed between these distributions at various stages. Comparing across stages, it is observed that in female cancers, a higher proportion of genes in early stages follow genextreme, GM, and dweibull distributions compared to later stages. Conversely, more genes in later stages exhibit gamma, $\chi ^{2}$, exponpow, and expon distributions. In male cancers, distribution patterns between early and later stages show less obvious distinctions across most distributions. However, both female and male cancers in the early stages tend to have a higher representation of genes following GM distributions compared to the later stages.

#### Limited occurrence of genes following normal distributions in large-scale transcriptomic data

Comparing the fitted distributions between cancer and normal samples, it is observed that 11,146 genes in cancer samples can be optimally fitted with one of the 16 distributions, predominantly following log-norm, genextreme, and GM distributions. In contrast, for normal samples, 15,455 genes can be optimally fitted with a distribution, mainly encompassing log-norm, GM, genextreme, gamma, $\chi ^{2}$, beta, and Pareto distributions. Notably, none of the genes in cancer samples are fitted with a normal distribution, while only 227 genes are optimally fitted with normal distributions in normal samples. This raises the question of whether normal distributions are rare in other large-scale omics data.

To investigate whether normal distributions are indeed rare, various datasets are examined (Table 3). Initially, disregarding health status, samples are divided by gender, resulting in 5,321 samples for males and 5,677 samples for females. Distribution fitting shows that 12,617 and 12,767 genes can be optimally fitted with one of the 16 distributions, yet none follows a normal distribution. Next, considering health status, analysis of 4,969 male and 5,307 female cancer samples also reveal no genes following a normal distribution. However, among 352 male and 370 female normal samples, 395 and 839 out of 19,156 genes respectively are optimally fitted with normal distributions. Further examination of racial groups included 655 Asian, 927 Black, and 7,758 White cancer samples, where none of the genes follows a normal distribution. Conversely, among 590 White and 53 Black normal samples, 456 and 454 genes respectively are optimally fitted with normal distributions.

**Table 3:** The number of genes that follow normal distributions in various datasets.

Dataset	Norm.	Can.	Male	Fem.	Male nor.	Male can.	Fem. nor.	Fem. can.	Asian can.	Black can.	Black nor.	White can.	White nor.
Samp. size	740	10512	5321	5677	352	4969	370	5307	655	927	53	7758	590
Fit. genes	15455	11146	12617	12767	16037	12607	16089	12739	14952	15104	13556	11888	13559
Norm. distr.	227	0	0	0	395	0	839	0	0	0	454	0	456
Prop.	1.19%	0	0	0	2.06%	0	4.38%	0	0	0	2.37%	0	2.38%

In summary, normal distributions are scarce in large-scale omics data. Across the datasets considered, fewer than 4.5% of genes follow normal distributions in normal samples, with small sample sizes potentially contributing to the appearance of normal distributions (Table 3). Decreased sample sizes reduce the impact of noise and outliers, significantly reducing the number of unfitted genes, which may allow for a few genes to exhibit normal distributions.

### Identifying informative genes via skewness of gene distributions

#### Identifying DEGs distinguishing cancer and normal samples using skewness ratio

**Figure 6:**
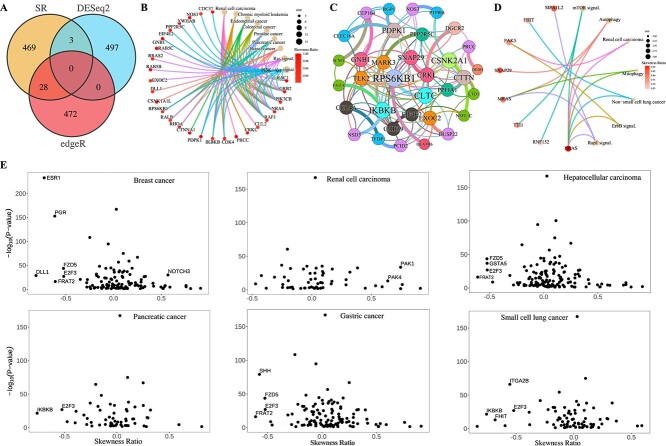
**Performances of the $SR$ in identifying DEGs. A**. Venn diagram for DEGs screened by $SR$, DESeq2, edgeR when comparing between cancer and normal samples. **B**. KEGG enrichment analysis for the top 469 genes (Not identified in the top 500 by DESeq2 and edgeR) that are uniquely identified by $SR$ when comparing between cancer and normal samples. Significantly enriched pathways ($P<0.05$) related to cancers are displayed. Similarly hereinafter. **C**. Network analysis of the 469 genes uniquely selected by $|SR|$. Thin links indicate interactions curated from the STRING database, while thick links represent connections also predicted by the partial $KCC$ (absolute partial correlation greater than 0.1). **D**. KEGG enrichment analysis for the top 93 genes that are uniquely identified by $SR$ when comparing between male and female cancer samples. **E**. The $SR$ versus $P$ values from the Wilcoxon test for genes that involve in the breast cancer, renal cell carcinoma, hepatocellular carcinoma, pancreatic cancer, gastric cancer, and small cell lung cancer pathways. Here, only genes with $P<0.05$ are considered, and names of genes with $-log_{10}(P)>10$ and $|SR|>0.5 $ are displayed.

Recognizing skewed gene distributions in cancer datasets and variations across different datasets, a statistical measure called the Skewness Ratio ($SR$) based on skewness [68] and the Wilcoxon rank-sum test [50] is proposed (**Materials and Methods**). The $SR$ facilitates assessment of skewness variations in gene expression profiles between datasets. Based on the proposed $SR$ metric, and the expression profiles of the 19,156 genes across 10,152 cancer samples and 730 normal samples, 16,492 genes show significant differential expression with $|SR|>0.5$ and $P<0.05$ (Wilcoxon rank-sum test). Notably, well-known oncogenes such as FAT4, KMT2C, KRAS, and PIK3CA display high $|SR|$ values of 0.7062, 0.5315, 0.9152, and 0.7746, respectively, indicating highly skewed distributions. To validate the biological relevance of genes with high $|SR|$ and to compare their identification with traditional methods like DESeq2 and edgeR [41, 42], the top 500 genes identified by the three methods are examined. Among these, 469 genes are uniquely identified by $SR$, and notably, 17 of them are confirmed as canonical cancer drivers [59]. KEGG enrichment analysis reveals that these 469 genes are significantly enriched in cancer-related pathways, including Ras signaling, PI3K-Akt signaling, and Renal cell carcinoma pathways (Fig. 6A, Supplementary Material and Fig. S1).

Based on the STRING database [48] and using the partial Kendall correlation coefficient ($KCC$) [69], a network is constructed for the 469 genes uniquely identified via $|SR|$ (Fig. 6B, Supplementary material and Fig. S2). Figure 6B highlights several gene pairs connected by protein–protein interactions and predicted by partial correlations. Notably, interactions include MARK3 and PPP2R5C ($KCC = 0.1468$), FNTA and IKBKB ($KCC = 0.2028$), NSD3 and IKBKB ($KCC = 0.1154$), HEATR6 and RPS6KB1 ($KCC = 0.1333$), CLTC and RPS6KB1 ($KCC = 0.1158$), DUSP22 and EXOC2 ($KCC = 0.1209$), SNAP29 and CRKL ($KCC = 0.1409$), CRKL and DGCR2 ($KCC = 0.1058$), E1F4E2 and CAB39 ($KCC = 0.1032$), GNB1 and CEP104 ($KCC = 0.1922$), PTPRB and NOS3 ($KCC = 0.1950$), RGP1 and PDPK1 ($KCC = 0.1072$), CLEC16A and PDPK1 ($KCC = 0.1020$), DEDD and PRCC ($KCC = 0.1760$). Figure 6A illustrates that PPP2R5C, IKBKB, RPS6KB1, EXOC2, CRKL, EIF4E2, GNB1, NOS3, PDPK1, and PRCC are involved in multiple cancer pathways, suggesting that MARK3, FNTA, HEATR6, CLTC, DUSP22, SNAP29, DGCR2, CAB39, CEP104, NSD3, PTPRB, RGP1, CLEC16A, DEDD, and TLK2 may also play significant roles in these cancers.

#### Identifying DEGs distinguishing between female and male cancer patients using skewness ratio

Comparing the 5,307 female cancer samples with the 4,969 male cancer samples, 15,406 genes that exhibit significant skewness differences ($|SR|>0.5$ and $P<0.05$) are identified. As discussed in the former subsection, genes EIF1AY, IGF1R, KDM5D, NRAS, PPP6C, and UTY have been previously reported to demonstrate gender differences in lung cancer, gastric cancer, liver cancer, melanoma, and acute myeloid leukemia. The skewness ratio ($SR$) values for these genes are 0.8923, 0.7595, 0.8302, 0.9112, 0.8111, and 0.8663, respectively, indicating substantial variations in skewness between the female and male cancer datasets.

The top 500 DEGs identified by DESeq2, edgeR, and $SR$ are further analyzed. Among these, $SR$ uniquely identified 93 genes, which show significant enrichment in several cancer pathways such as non-small cell lung cancer and renal cell carcinoma (Fig. 6C, Supplementary material and Fig. S3). Previous studies have indicated gender differences in these types of cancers [70, 71].

Among the 15,406 genes exhibiting significant skewness differences between female and male cancer samples, those involved in breast cancer, gastric cancer, hepatocellular carcinoma, pancreatic cancer, renal cell carcinoma, and small cell lung cancer pathways are specifically examined. Scatter plots depicting $SR$ versus $-log_{10}$($P$) for these pathway-associated genes are presented in Fig. 6D–I. Our findings illustrate that each pathway includes several genes displaying notable skewness differences between female and male patients, suggesting these genes could potentially serve as important markers for gender prediction in samples.

### Improved naïve Bayes integrating gene-specific distributions for sample classification

#### Performance of the improved naïve Bayes in simulated data

By integrating gene-specific distributions into the traditional NB framework [72], we propose an improved NB (INB) classifier for sample classification (**Materials and Methods**). To validate the effectiveness of the INB model in simulated data, we conduct two simulations using randomly generated datasets with specific distributions.

Firstly, we explored the computational processes of the INB model through two simple examples, where only two variables with specific distributions are considered (detailed in the Supplementary material). These examples revealed that the traditional NB model can suffer from compromised classification accuracy when incorrect distribution assumptions are made. Conversely, the INB model demonstrated superior performance in handling categorical data with diverse distributions.

Secondly, two datasets, each containing 400 samples and 200 variables, are generated to represent two distinct sample types. Within each dataset, 100 variables follow a generalized extreme value distribution, while the remaining 100 variables follow a log-normal distribution. These datasets served as training sets for the model. Subsequently, 80 samples are generated as test sets. To compare the performance of the INB and the traditional NB, the actual distribution for each variable is utilized in the INB model, while the NB model assumes all variables followed normal distributions. The $F1$-score ($F1$) and $Youden$ index ($YI$) for each method are averaged over 50 random simulation runs. Results indicate that the INB achieves superior classification performance, with an average $F1$ of 0.9774 and a $YI$ of 0.9548, compared to the NB’s average $F1$ of 0.8536 and $YI$ of 0.71. This clearly demonstrates the benefit of incorporating gene-specific distributions in the INB model. Notably, the experiment also reveals that even when datasets do not conform to normal distributions, the NB model still exhibits reasonable classification performance.

#### Performance of the improved naïve Bayes in sample classification

To assess the classification performance of the INB in real-world datasets, datasets for hepatocellular carcinoma (HCC), breast cancer (BRCA), kidney cancer (KICA), lung adenocarcinoma with lung squamous cell carcinoma (LUAD_LUSC), and thyroid carcinoma with bladder urothelial carcinoma (THCA_BLCA) are considered (**Materials and Methods**). These datasets include specific cancer samples alongside their corresponding normal samples, as well as datasets with mixed cancer samples. For datasets with specific cancer samples, features in the classifiers are derived from KEGG pathway genes associated with the respective cancers. In contrast, for datasets with mixed cancer samples (LUAD_LUSC and THCA_BLCA), the feature set includes the union of KEGG pathway genes corresponding to both cancer subtypes. It’s important to note that in the HCC, BRCA, and KICA datasets, the cancer samples represent a subset of specific cancer cases, with normal samples typically sourced from healthy tissues of corresponding cancer patients. Conversely, in the LUAD_LUSC and THCA_BLCA datasets, samples are sourced from different patients.

To validate the advantages of using KEGG pathway genes and to assess the classification performance of the INB using gene-specific distributions, two control experiments are conducted: 1) INBFD: The INB using only five types of distributions (expon, pareto, log-gamma, uniform, and normal distributions). 2) INBRSG: Randomly selecting an equivalent number of genes from the dataset for sample classification. Additionally, the Support Vector Machine (SVM) and Multilayer Perceptron (MLP) classifiers are included for comparison [73, 74]. For each experiment, 80% and 60% of randomly extracted samples are used as training datasets, while the remaining samples serve as the testing dataset.

**Figure 7:**
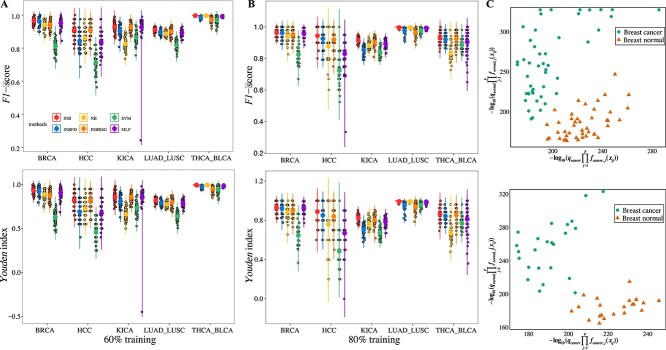
**Performances of the INB classifier in sample classification. A**. $F1$ and $YI$ for the INB, NB, INBFD, INBRSG, SVM, and MLP using 60% training data across the five datasets. **B**. $F1$ and $YI$ for the INB, NB, INBFD, INBRSG, SVM, and MLP using 80% training data across the five datasets. **C**. Sample classification using INB in the breast cancer dataset with 60% training data and 80% training data. Here, $q_{k}\prod _{j=1}^{p}f_{kj}(x_{ij})$ is transformed using $-log_{10}$.

The average $F1$ and $YI$ from 50 simulation runs indicate that the INB outperforms the NB across the five datasets. In all cases, the $F1$ score of the INB exceeds 0.91 and the $YI$ surpasses 0.82 (see Table 4, Fig. 7A and B). Notably, in the KICA dataset with 80% training data, the INB achieves an $F1$ score nearly $12\%$ higher than that of the NB, and the $YI$ is 28% higher. Scatter plots of $-log_{10}(q_{k}\prod _{j=1}^{p}f_{kj}(x_{ij}))$ for breast cancer and normal datasets illustrate that the INB effectively distinguishes between cancer and normal samples. Comparisons among INB, NB, INBFD, INBRSG, SVM, and MLP (Table 4 and Fig. 7C) consistently show that the INB maintains superior performance in sample classification, underscoring the benefit of using accurate gene-specific distributions. In summary, the proposed INB, which incorporates optimal gene-specific distributions, demonstrates superior classification performance compared to the NB, SVM, and MLP.

**Table 4:** Mean values and standard deviations of $F1$ and $YI$ with 80% and 60% training data in the five datasets based on 50 random simulation runs.

Five methods or control experiments are considered, including NB, INBFD, INBRSG, SVM, and MLP.													
	Dataset	80% training						60% training					
		INB	NB	INBFD	INBRSG	SVM	MLP	INB	NB	INBFD	INBRSG	SVM	MLP
	BRCA	**0.9647**	0.9421	0.9617	0.9515	0.8246	0.9547	**0.9670**	0.9417	0.9625	0.9392	0.8126	0.9518
		(0.0245)	(0.0340)	(0.0219)	(0.0278)	(0.0479)	(0.0288)	(0.0196)	(0.0249)	(0.0225)	(0.0284)	(0.0367)	(0.0284)
	HCC	**0.9437**	0.8773	0.9252	0.9195	0.7235	0.8299	**0.9106**	0.8619	0.8410	0.9075	0.7121	0.8358
$F1$		(0.0515)	(0.0975)	(0.0586)	(0.0532)	(0.0901)	(0.1011)	(0.0436)	(0.0494)	(0.0551)	(0.0438)	(0.0556)	(0.0603)
	KICA	**0.9314**	0.8317	0.9211	0.9258	0.8847	0.9038	**0.9287**	0.8263	0.9047	0.9278	0.8661	0.9269
		(0.0336)	(0.0474)	(0.0291)	(0.0399)	(0.0318)	(0.0734 )	(0.0294)	(0.0344)	(0.0304)	(0.0265)	(0.0351)	(0.1020)
	LUAD_LUSC	**0.9133**	0.8822	0.8536	0.9029	0.8376	0.8932	**0.9107**	0.8877	0.8965	0.8990	0.8200	0.8917
		(0.0174)	(0.0218)	(0.0261)	(0.0261)	(0.0258)	(0.0218)	(0.0137)	(0.0143)	(0.0144)	(0.0230)	(0.0259)	(0.0171)
	THCA_BLCA	**0.9959**	0.9958	0.9772	0.9655	0.9758	0.9919	**0.9967**	0.9963	0.9860	0.9727	0.9712	0.9915
		(0.0049)	(0.0043)	(0.0213)	(0.0251)	(0.0144)	(0.0068)	(0.0029)	(0.0028)	(0.0088)	(0.0158)	(0.0214)	(0.0047)
	BRCA	**0.9286**	0.8864	0.9225	0.9006	0.6474	0.9096	**0.9335**	0.8850	0.9239	0.8762	0.6261	0.9039
		(0.0504)	(0.0668)	(0.0589)	(0.0571)	(0.0916)	(0.0576)	(0.0397)	(0.0494)	(0.0460)	(0.0579)	(0.0694)	(0.0567)
	HCC	**0.8880**	0.7600	0.8520	0.8400	0.486	0.67	**0.8220**	0.7260	0.6880	0.8160	0.458	0.674
$YI$		(0.1023)	(0.1895)	(0.1147)	(0.1050)	(0.1539)	(0.1693)	(0.0864)	(0.0970)	(0.1052)	(0.0866)	(0.0966)	(0.1192)
	KICA	**0.8638**	0.6728	0.8431	0.8531	0.7722	0.8124	**0.8584**	0.6620	0.8114	0.8561	0.7358	0.8553
		(0.0660)	(0.0885)	(0.0575)	(0.0780)	(0.0618)	(0.1369)	(0.0577)	(0.0637)	(0.0590)	(0.0525)	(0.0678)	(0.1950)
	LUAD_LUSC	**0.8256**	0.7633	0.7067	0.8049	0.6731	0.7864	**0.8207**	0.7738	0.7934	0.7970	0.6379	0.7836
		(0.0350)	(0.0438)	(0.0519)	(0.0525)	(0.0506)	(0.0430)	(0.0277)	(0.0288)	(0.0283)	(0.0458)	(0.0499)	(0.0337)
	THCA_BLCA	**0.9926**	0.9921	0.9532	0.9368	0.9564	0.9833	**0.9940**	0.9930	0.9742	0.9500	0.9474	0.9827
		(0.0089)	(0.0079)	(0.0445)	(0.0462)	(0.0260)	(0.0142)	(0.0052)	(0.0053)	(0.0164)	(0.0288)	(0.0388)	(0.0099)

#### Performance of the improved Naïve Bayes in other testing data

Based on the previously trained INB classifiers from the breast cancer and kidney cancer datasets in the aforementioned section, some extra data for the two cancers are explored. The extra data includes the previously unused samples from the TCGA for the two cancer, as well as the breast cancer dataset GSE202203 from NCBI [62]. The unused TCGA breast cancer data consist of 1,002 cancer samples and 3 normal samples. The unused TCGA kidney cancer data include 773 cancer samples and 3 normal samples. The GSE202203 dataset contains 3,207 samples for breast cancer.

For the extra TCGA breast cancer samples, the INB achieves an $F1$ score of 0.9882 and a $YI$ of 0.9810, while the NB method achieves an $F1$ score of 0.9574 and a $YI$ of 0.9232. In the breast cancer dataset GSE202203 from NCBI, the INB achieves an $F1$ score of 0.9992, surpassing the NB’s $F1$ score of 0.9782. Regarding the extra TCGA kidney cancer samples, the INB achieves an $F1$ score of 0.9955 and a $YI$ of 0.6628, compared to the NB’s $F1$ score of 0.8275 and a $YI$ of 0.7102. Although the $YI$ of the INB is slightly lower than that of the NB for the extra TCGA kidney cancer samples, the $F1$ score of the INB is significantly higher, underscoring the practical efficacy of the INB. Despite relying solely on the fitted distributions of genes within associated KEGG pathways, the INB notably enhances classification accuracy.

## Discussion

Cancer remains a formidable global health challenge with significant mortality rates. Current research often assumes that genes conform to singular distributions such as normal, multinomial, or Bernoulli distributions. However, the true distributions of genes in large-scale omics data, including their variations across genders and racial groups, are not well understood. Advances in high-throughput sequencing have facilitated the exploration of gene expression distributions across different datasets derived from TCGA, categorized by cancer types, racial demographics, and gender. Rigorous fitting criteria, including $BIC$ and KS tests, reveal distinct distribution patterns among known cancer driver genes, gender-specific genes, and racial-specific genes. Notably, a majority of genes exhibit highly skewed distributions, with cancer samples preferring genextreme, log-norm, and GM distributions, while normal samples show preferences for gamma, beta, $\chi ^{2}$, Pareto, exponpow, and expon distributions. Importantly, over 95.5% of genes deviate from normal distributions, a trend particularly pronounced in larger cancer datasets.

Investigations into gene expression heterogeneity show that cancer samples exhibit more asymmetric distributions compared to their normal counterparts. For instance, while only 275 genes in normal samples display symmetric distributions (227 normal distributions, 38 $t$ distributions, and 10 Laplace distributions), this pattern is absent in all cancer samples, including female and male subsets. These findings underscore how increased heterogeneity in gene expression profiles contributes to distinct distribution patterns in cancer versus normal samples.

Further exploration includes comparing gene distributions between bulk and scRNA-seq datasets. Notably, the majority of genes in scRNA-seq datasets cannot be fitted to any of the 16 distributions considered. This discrepancy is attributed to the “dropout” phenomenon in scRNA-seq, where a gene may be expressed in some cells but undetected in others due to low RNA input and stochastic gene expression.

Motivated by these findings on gene distribution diversity, a new metric called $SR$ is introduced to measure skewness variation across datasets. This metric evaluates gene importance in distinguishing between datasets based on their distributional deviations. Additionally, the Wilcoxon test verifies significant expression differences for the considered genes between datasets. The $SR$ provides a holistic view of gene significance, contrasting with traditional methods like $\log _{2}(FC)$, which only assess average expression differences and are susceptible to outlier impacts. Numerical simulations across cancer and normal datasets demonstrate that top-ranked genes by $SR$ frequently include canonical cancer drivers and are enriched in cancer-related pathways. Furthermore, in gender-specific cancer analyses, $SR$ effectively identifies DEGs associated with gender-biased cancers.

To harness the practical utility of gene-specific distributions, an INB classifier is developed. Unlike traditional NB, which assumes genes follow standard distributions, INB fits optimal distributions for each gene across datasets, enhancing classification accuracy. The methodological approach of leveraging variable-specific distributions can be extended to other algorithms to bolster their classification capabilities. Future research could also explore additional distributions beyond the 16 distributions, such as the skew generalized normal distribution [75]. Additionally, developing methods that relax the independence assumption in the NB and INB models [76, 77] presents a promising avenue for future work. Our method involves fitting multiple distribution models, while applying MLE and the EM algorithm to optimize the parameters of these distributions. These computational processes, especially when dealing with large-scale gene expression data, may require considerable computational resources. For INB, running a simulated dataset with 20 samples and 10,000 variables on an Intel(R) Core (TM) i7-8700 CPU @ 3.20GHz 3.19 GHz takes 8,583 seconds. Although the computational time is manageable for ordinary computers, to further improve computational efficiency, several optimization strategies can be considered, such as vectorized operations, parallel computing, and a distribution pre-screening strategy. Additionally, although our method primarily relies on CPU computation, for larger datasets, it may be beneficial to migrate some computational tasks to GPU, particularly in terms of parallelization and matrix operations, where GPU computing can significantly enhance efficiency.

Challenges and avenues for future investigation include exploring the performance of $SR$ when using fitted gene distributions, addressing the computational complexity of INB for large gene sets by focusing on KEGG pathway genes, and integrating gene network information to refine classification algorithms. Beyond differential expression and sample classification, future research could delve into inferring gene regulatory networks [78]. Moreover, expanding this study to encompass multi-omics data promises further insights, which is a direction to be pursued in future work.

## Conclusions

This paper deciphers gene distributions in large-scale RNA-seq data and explores their applications. Extensive statistical analysis reveals that genes in normal samples exhibit more diverse distributions compared to those in cancer samples. Genes in cancer samples tend to prefer distributions such as genextreme, log-norm, and GM, whereas genes in normal samples favor gamma, beta, $\chi ^{2}$, Pareto, exponpow, and expon distributions. Remarkably, less than 4.5% of genes in normal samples follow normal distributions. Some well-known cancer drivers and genes with gender and racial differences exhibit distinct distribution characteristics across datasets. Based on the differences in gene skewness between two datasets, a metric called $SR$ is introduced, which provides an alternative method to explore DEGs. Numerical simulations across multiple datasets demonstrate that the $SR$ measure offers advantages over traditional methods like DESeq2 and edgeR. Furthermore, an INB classifier is developed, which incorporates gene-specific distributions and shows higher classification accuracy and stability compared to the traditional NB classifier in various datasets. These investigations not only deepen our understanding of gene distribution patterns but also introduce novel approaches for DEG screening and sample classification.

**Key Points**

- Gene expression patterns in large-scale transcriptomic data are deciphered.
- A novel skewness-based metric is proposed to screen differentially expressed genes across two datasets.
- An improved naïve Bayes method incorporating gene-specific distributions can well realize sample classification.

## Supplementary Material

BIB-24-1346FinalVersion-Supplementary_material_bbae590
