# Supplementary material for: Deciphering gene expression patterns using large-scale transcriptomic data and its applications
Source: Brief Bioinform. 2024 Nov 14;25(6):bbae590. doi: 10.1093/bib/bbae590 (PMC11562847; doi:10.1093/bib/bbae590)
Supplement: BIB-24-1346FinalVersion-Supplementary_material_bbae590 [file bib-24-1346finalversion-supplementary_material_bbae590.docx]

**Supplementary materials: Deciphering gene expression patterns using large-scale transcriptomic data and its applications**

**Shunjie Chen^1^, Pei Wang^1, 2^*****, Haiping Guo^1^, Yujie Zhang^1^**

^1^School of Mathematics and Statistics, Henan University, Kaifeng 475004, Henan, China.

^2^Henan Engineering Research Center for Industrial Internet of Things, Henan University, Zhengzhou 450046, China.

*To whom correspondence should be addressed; Email: [wangpei@henu.edu.cn](mailto:wangpei@henu.edu.cn)

**This PDF file includes:**

Supplementary Text

Figs. S1 to S3

Table S1

**1. Further details on fitting gene distributions**

***1.1. Classification of the 16 distributions and their relationships***

The 16 distributions considered in this paper can be grouped via the shape, symmetry, and modality of probability density functions (PDFs).

Based on the shape of PDFs, distributions can be divided into light-tailed and heavy-tailed distributions. Light-tailed distributions, such as the normal distribution, exponential distribution, and beta distributions under certain parameters, have rapidly decaying tails. Heavy-tailed distributions, including the Cauchy distribution, Laplace distribution, log-normal distribution, and gamma distributions under certain parameters, have slowly decaying tails and a higher probability of extreme values.

Based on symmetry of PDFs, distributions can be divided into symmetric and asymmetric. Symmetric distributions, like the normal distribution, Laplace distribution, and student’s *t* distribution (with large degrees of freedom), have density functions that are symmetric about the central point. Asymmetric distributions, such as the exponential distribution, gamma distribution, log-normal distribution, Gaussian mixture (GM) distribution and beta distributions under some parameters, have PDFs that are not symmetric.

Based on modality of PDFs, distributions can be divided into unimodal and multimodal ones. Unimodal distributions, including the normal distribution, Laplace distribution, exponential power, gamma, beta, generalized extreme value, Cauchy, $\chi^{2}$, log-normal, log-gamma, and student’s *t* distribution, have a single peak in their probability density curves. Multimodal distributions, like the GM distribution and the double Weibull distribution, can have multiple peaks.

By using these classification methods, one can more precisely analyze and understand the characteristics of different distributions/genes, thereby selecting the most appropriate parameter estimation methods. There are also some internal relationships among the 16 distributions, under certain parameters, these distributions are equivalent.

**1) Normal distribution and student’s t distribution**

For a small degree of freedom (DF) *m,* there are many differences between normal distributions and *t* distributions; With the increase of *m*, the PDFs of the *t* distribution approximate to normal distributions. As the DF *m* approaches infinity, the student's *t* distribution approaches the normal distribution.

**2) Normal distribution and logarithmic normal distribution**

If a random variable ln*(****X****)* follows normal distribution, then ***X*** follows a logarithmic normal distribution.

**3) Normal distribution and Gaussian mixture distribution**

A GM distribution is a weighted sum of multiple normal distributions. When only one normal distribution is considered, the GM distribution degenerates into the traditional normal distribution.

**4) Normal distribution and Laplace distribution**

Both normal and Laplace distributions are symmetric, but the Laplace distribution has heavier tails, meaning its tails decay slower than those of the normal distribution.

**5) Exponential distribution and gamma distribution**

The exponential distribution is a special case of the gamma distribution, when the shape parameter *α=*1 in the gamma distribution, the two distributions are equivalent.

**Derivation**: Let ***X*** ∼Gamma*(α, β)*, its PDF is:

$$f(x)=\frac{\beta^{\alpha}x^{\alpha-1}\exp(-\beta x)}{\Gamma(\alpha)}.$$

When *α=*1, the PDF degenerates into

$$f(x)=\beta\exp(-\beta x)\text{，}$$

which corresponds to the PDF of the exponential distribution.

**6) Beta distribution and uniform distribution**

The beta distribution becomes the uniform distribution when *α=β=1*.

**Derivation**: Let ***X****∼*Beta *(α, β)*, its PDF is:

$$f(x)=\frac{\Gamma(\alpha+\beta)x^{\alpha-1}(1-x)^{\beta-1}}{\Gamma(\alpha)\Gamma(\beta)}\text{，}\begin{matrix} & 0\leq x\leq1 \end{matrix}.$$

when *α=β=*1, it degenerates into:

$$f(x)=1\text{,}\begin{matrix} & 0\leq x\leq1 \end{matrix},$$

which is equivalent to the uniform distribution.

**7) Gamma distribution and $\boldsymbol{\chi}^{\mathbf{2}}$ distribution**

The gamma distribution becomes the $\boldsymbol{\chi}^{\mathbf{2}}$ distribution when the shape parameter is taken as *α=k/2* and the scale parameter is set as *β=1/*2.

**Derivation**: Let ***X***∼Gamma*(α, β)*, its PDF is:

$$f(x)=\frac{\beta^{\alpha}x^{\alpha-1}\exp(-\beta x)}{\Gamma(\alpha)},x>0.$$

When *α=k/2* and *β=1/*2:

$$f(x)=\frac{x^{\left(\frac{k}{2}-1\right)}\exp(-\frac{x}{2})}{2^{\frac{k}{2}}\Gamma\left(\frac{k}{2}\right)},x>0,$$

which is the PDF for the $\chi^{2}$ distribution.

**8) $\boldsymbol{\chi}^{\mathbf{2}}$ distribution and student’s t distribution**

The student’s *t* distribution is based on a normal distribution and a $\chi^{2}$ distribution that are independent with each other. Specifically, if ***X*** follows a standard normal distribution, ***Y*** follows a $\chi^{2}$ distribution with DF *m*, and the two distributions are independent with each other, then $t=\frac{X}{\sqrt{Y/m}}$follows a student’s *t* distribution with DF *m*.

**9) Beta distribution and gamma distribution**

The beta distribution can be obtained via two independent gamma-distributed random variables.

**Derivation**: Let *X*∼Gamma *(α,* 1*)*, *Y*∼Gamma *(β,* 1*)*, then $Z=\frac{X}{X+Y}$ follows Beta *(α, β).*

**10) Gamma distribution and log-gamma distribution**

If ln(***X***) follows a gamma distribution, then the random variable ***X*** follows a log-gamma distribution.

***1.2. Distribution fitting criteria and priority of considered distributions***

To fit gene expression distributions, two metrics are considered. Firstly, the Bayesian Information Criterion (BIC) is used to select a potentially optimal distribution, and then the Kolmogorov-Smirnov (*KS*) test is performed to determine whether the distribution can statistically fit the data. Each gene is fitted among the 16 distributions until the optimal distribution (which has the smallest BIC among the distributions with *KS* *p*-value>=0.01) is obtained. The detailed procedures are provided in Fig.1 of the main text.

The BIC formula is as follows:

$$BIC=-2\ln(L)+k\ln(n).$$

Here, *L* is the maximum value of the likelihood function for each distribution (For details, see **subsection 1.3 of the Supplementary material**); *k* is the number of parameters in the model, *n* is the sample size. BIC penalizes complex models in order to prevent overfitting. Even if a complex model fits the training data better, but if its complexity is too high, then its BIC score will increase, making it less preferable in model selection. Moreover, when comparing among different models, the one with the smallest BIC value is preferred, since a smaller BIC value indicates that the fitted distribution can well balance between the fitting performance and model complexity.

During fitting gene expression distributions, the BIC value of each model is firstly calculated to evaluate their performance, and then the model with the smallest BIC value and a *KS* test *P*-value >= 0.01 is chosen as the optimal distribution. This ensures that the selected model not only performs well in fitting the data but also has an acceptable level of complexity.

It is noted that, if the BIC values for two distributions are equal and are both the smallest, we automatically select the first distribution in predefined order as the optimal distribution. The priority for the 16 distributions during our fitting processes is defined as: GM, normal, student’s *t*, pareto, double Weibull, generalized extreme value, Laplace, Cauchy, $\chi^{2}$, exponential, exponential power, gamma, beta, log-normal, log-gamma, uniform. Such priority mainly considers the popularity of the 16 distributions in existing works.

***1.3. Parameter estimation methods***

For the normal, student’s *t*, Pareto, generalized extreme value, Laplace, Cauchy, $\chi^{2}$, exponential, exponential power, gamma, beta, log-normal, log-gamma, and uniform distributions, the Maximum Likelihood Estimation (MLE) is used to estimate model parameters; whereas, for the GM and double Weibull distributions, the Expectation Maximization (EM) algorithm is used to perform parameter estimation.

***1.3.1 Maximum likelihood estimation***

For MLE, taking the normal distribution as an example, detailed processes will be introduced as follows. Assume that gene$X_{i}\sim N(\mu,\sigma^{2})$, and given *n* independent observations, each observation $x_{ki}\sim N(\mu,\sigma^{2}),k=1,2,...,n.$ To estimate the mean$\mu$and the variance$\sigma^{2}$of the normal distribution, the likelihood function can be established as:

$L=\prod_{k=1}^{n} \frac{1}{\sqrt{2\pi}\sigma}\exp\left(-\frac{(x_{ki}-\mu)^{2}}{2\sigma^{2}}\right).$ (1)

In real-world applications, parameters can be estimated via minimizing the negative logarithmic likelihood function:

$$(\hat{\mu},\hat{\sigma}^{2})=\mathrm{argmin}_{\mu,\sigma^{2}}\{-\ln L\}=\mathrm{argmin}_{\mu,\sigma^{2}}\left\{\frac{n}{2}\ln(2\pi\sigma^{2})+\frac{1}{2\sigma^{2}}\sum_{k=1}^{n} (x_{ki}-\mu)^{2}\right\}.$$

After calculating the first order partial derivative of$-\ln L$with respect to the two parameters, and setting them as zero, we obtain:

$$\hat{\mu}=\bar{x}_{i}=\frac{1}{n}\sum_{k=1}^{n} x_{ki}.$$

$$\hat{\sigma}^{2}=s_{i}^{2}=\frac{1}{n}\sum_{k=1}^{n} (x_{ki}-\bar{x}_{i})^{2}.$$

For the other distributions, parameters can be similarly estimated via the above procedures.

***1.3.2. Expectation Maximization***

For the EM method, we take the GM distribution as an example. The PDF of a GM distribution consists of the weighted sum of *K* (*K*>=1) Gaussian distributions (normal distributions), which can be written as:

$$f(x)=\sum_{k=1}^{K} w_{k}\frac{1}{\sqrt{2\pi}\sigma_{k}}\exp\left(-\frac{(x-\mu_{k})^{2}}{2\sigma_{k}^{2}}\right),w_{k}\in(0,1),\sum_{k=1}^{K} w_{k}=1.$$

Here,$\mu_{k}$is the mean and$\sigma_{k}^{2}$is the variance for the *k*’th normal distribution,$w_{k}$is the weight.

For the observation vector$\text{X}_{i}=(x_{1i},x_{2i},...,x_{ni})^{T}$of gene *i*, to fit a GM distribution for gene *i*, one needs to determine the parameters$\mu_{k}$,$w_{k}$and$\sigma_{k}$. Traditionally, the EM method is frequently used to estimate these parameters. Suppose there is a latent variable$\gamma_{tk}$that denotes the posterior probability of data point$x_{ti}$belonging to the *k*’th Gaussian component. The EM method firstly initialize the parameters$\mu_{k}$,$w_{k}$and$\sigma_{k}$, and then iteratively performs the following E-step and M-step.

**E-step:** Calculate the posterior probability$\gamma_{tk}$of$x_{ti}$belonging to the *k*’th Gaussian component, and denote it as:

$$\gamma_{tk}=\frac{w_{k}N(x_{ti};\mu_{k},\sigma_{k})}{\sum_{j=1}^{K} w_{j}N(x_{ti};\mu_{j},\sigma_{j})},t=1,2,...,n;k=1,2,...,K.$$

The variable$\gamma_{tk}$describes the responsibilities of each component for each observation. The expected log-likelihood for the GM distribution can now be written as:

$Q(\theta)=\ln L=\sum_{t=1}^{n} \sum_{k=1}^{K} \gamma_{tk}\ln\left[w_{k}N(x_{ti};\mu_{k},\sigma_{k})\right]=\sum_{t=1}^{n} \sum_{k=1}^{K} \gamma_{tk}\left[\ln w_{k}-\ln(\sqrt{2\pi}\sigma_{k})-\frac{(x_{ti}-\mu_{k})^{2}}{2\sigma_{k}}\right].$ (2)

Here,$\theta$is a set that represents all the parameters in the model, and the function$Q(\theta)$is the weighted sum of the log-likelihoods of all data points under each Gaussian component.

**M-step:** Update the parameters$\theta$(means, variances, and mixture coefficients) of the GM to maximize$Q(\theta)$in the E-step. Taking the first-order derivative of$Q(\theta)$with respect to parameters$\mu_{k},\sigma_{k},w_{k}(k=1,2,...,K)$and setting them to be 0, one can obtain:

$$\hat{\mu}_{k}=\frac{\sum_{t=1}^{n} \gamma_{tk}x_{ti}}{\sum_{t=1}^{n} \gamma_{tk}},$$

$$\hat{\sigma}_{k}^{2}=\frac{\sum_{t=1}^{n} \gamma_{tk}(x_{ti}-\hat{\mu}_{k})^{2}}{\sum_{t=1}^{n} \gamma_{tk}},\hat{w}_{k}=\frac{\sum_{t=1}^{n} \gamma_{tk}}{n}.$$

After obtaining the estimation$\hat{\mu}_{k}$,$\hat{\sigma}_{k}^{2}$,$\hat{w}_{k}$, the algorithm returns to the E-step to recompute$\gamma_{tk}$, and subsequently performs the M-step to iteratively estimate$\mu_{k}$,$w_{k}$and$\sigma_{k}$until they converge or until a predefined step of iterations is achieved.

It is noted that the function *L* in Eqs.(1) or (2) is employed to compute the BIC.

***1.4. Data processing during gene distribution fitting***

For the observation vector of the *i*’th gene $\text{X}_{i}=(x_{1i},x_{2i},...,x_{ni})^{T}$, since different distributions may have different requirements on$x_{ti}$, data processing is essential for some distributions.

For the distributions such as normal, student’s *t*, double Weibull, generalized extreme value, Laplace, Cauchy, $\chi^{2}$, exponential, exponential power, gamma, log-norm, log-gamma, uniform, and GM distributions, the TPM values are always nonnegative, there is no need to perform data processing.

The beta distribution requires *0≤x≤1*, the following normalization process will be performed for data when fitting the beta distribution:

$$x_{_{ti}}^{*}=\frac{x_{ti}-\min(X_{i})}{\max(X_{i})-\min(X_{i})},t=1,2,...,n.$$

***1.5. Statistical Significance Analysis of Genes with Different Datasets***

For the sections "Distributions of cancer driver genes exhibit notable differences between cancerous and normal tissues," " Gender-based differences in gene distributions" and " Gene distributions exhibit racial disparities," we performed Wilcoxon test to analyze the statistical significance of the genes with distributional differences that were highlighted in the paper. The analysis was conducted across different sample groups, and the results are shown in the following Supplementary Table S1:

**Supplementary Table S1. Wilcoxon test P-values for gene expression comparisons across different groups.**

| **Gene** | **Cancerous vs. Normal** | **Male vs. Female** | **White vs. Asian** | **White vs. Black** | **Asian vs. Black** | **TC. BRCA vs. TCGA_GSE202203 BRCA** |
| --- | --- | --- | --- | --- | --- | --- |
| **FAT4** | 3.18 × ${10}^{-129}$ | -- | -- | -- | -- | -- |
| **KMT2C** | 8.49 ×${10}^{-4}$ | -- | -- | -- | -- | -- |
| **KMT2D** | 0.1884 | -- | -- | -- | -- | -- |
| **PIK3CA** | 0.0318 | 3.36 ×${10}^{-5}$ | -- | -- | -- | -- |
| **KRAS** | 6.36 ×${10}^{-14}$ | -- | -- | -- | -- | -- |
| **PTEN** | 1.81 ×${10}^{-29}$ | -- | -- | -- | -- | -- |
| **NF1** | -- | 0.1052 | -- | -- | -- | -- |
| **EIF1AY** | -- | 0 | -- | -- | -- | -- |
| **IGF1R** | -- | 5.67 ×${10}^{-6}$ | -- | -- | -- | -- |
| **NRAS** | -- | 0.0075 | -- | -- | -- | -- |
| **KDM5D** | -- | 0 | -- | -- | -- | -- |
| **UTY** | -- | 0 | -- | -- | -- | -- |
| **PPP6C** | -- | 1.60 ×${10}^{-18}$ | -- | -- | -- | -- |
| **CTNNB1** | -- | -- | 1.00 ×${10}^{-24}$ | 0.0160 | 8.35 ×${10}^{-12}$ | -- |
| **ATM** | -- | -- | 1.11 ×${10}^{-17}$ | 1.15 ×${10}^{-5}$ | 4.22 ×${10}^{-5}$ | -- |
| **CHEK2** | -- | -- | 0.6381 | 7.76 ×${10}^{-9}$ | 9.67 ×${10}^{-5}$ | -- |
| **NCOR1** | -- | -- | 0.0004 | 0.00028 | 0.4774 | -- |
| **BRCA1** | -- | -- | -- | -- | -- | 9.68 ×${10}^{-11}$ |
| **BRCA2** | -- | -- | -- | -- | -- | 1.18 ×${10}^{-163}$ |

**2. Network analysis for the top-500 genes that are identified by SR**

***2.1. Methods to construct the gene co-expression network***

The partial Kendall correlation coefficient (KCC) is used to construct co-expression network for the top-500 genes identified by SR. The partial KCC is based on the KCC, which is a non-parametric statistic used to measure the ordinal association between two variables. The KCC is based on the ranks of the data and calculates the difference between the number of concordant and discordant pairs. Given two *n* dimensional observation vectors *X_i_* and *X_j_*, the KCC is defined as:

$$\tau_{ij}=\frac{n_{c}-n_{d}}{\sqrt{(n_{0}-n_{i})(n_{0}-n_{j})}}.$$

- $n_{0}=\frac{n(n-1)}{2}$ is the total number of pairs.
- $n_{i}=\sum_{l} \frac{x_{li}(x_{li}-1)}{2}$is the number of tied pairs only in *X_i_*.
- $n_{j}=\sum_{l} \frac{x_{lj}(x_{lj}-1)}{2}$is the number of tied pairs only in *X_j_*.
- $n_{c}$is the number of concordant pairs.
- $n_{d}$is the number of discordant pairs.

The partial KCC is defined as:

$$\tau_{ij.k}=\frac{\tau_{ij}-\tau_{ik}\tau_{jk}}{\sqrt{(1-\tau_{ik}^{2})(1-\tau_{jk}^{2})}}.$$

Here,$\tau_{ij}$ is the KCC between *X_i_* and *X_j_*,$\tau_{ik}$represents the KCC between *X_i_* and the third vector *X_k_*,$\tau_{jk}$ is the KCC between *X_j_* and the third vector *X_k_*.

***2.2. More details on enrichment analysis***

This subsection gives more details of the GO and KEGG enrichment analysis on the identified DEGs via SR (|SR|>0.5 and *P*-value<0.05).

***2.2.1. Enrichment analysis for the identified DEGs between cancer and normal samples***

Based on the observations of each gene in cancer and normal samples, the SR uniquely identified 469 genes that are in the top-500 ranking list. KEGG enrichment analysis (Fig.S1) reveals that the 469 genes involve in multiple cancer-related pathways, such as "Renal cell carcinoma (ko05211)", "Colorectal cancer (ko05210)", "Pancreatic cancer (ko05212)", "Chronic myeloid leukemia (ko05220)", "Endometrial cancer (ko05213)", "Acute myeloid leukemia (ko05221)", "Ras signaling pathway (ko04014)", "Non-small cell lung cancer (ko05223)". GO enrichment analysis (Fig.S1) further highlights biological processes associated with cancer. For instance, "Ubiquitin-dependent protein catabolic process" (GO:0006511) and "modification-dependent protein catabolic process" (GO:0019941) are involved in the degradation of proteins via the ubiquitin-proteasome system. Dysregulation in these pathways can lead to the accumulation of damaged or misfolded proteins, contributing to cancer development. "RNA processing" (GO:0006396) and "RNA binding" (GO:0003723) are crucial for the maturation and modification of RNA molecules, and their dysregulation can result in abnormal gene expression patterns observed in various cancers. "Cellular protein metabolic process" (GO:0044267) and "protein metabolic process" (GO:0019538) involve the synthesis, folding, and degradation of proteins, essential for maintaining cellular homeostasis. Disruptions in these processes can lead to tumorigenesis. "Intracellular transport" (GO:0046907) is vital for the proper localization of proteins and organelles, and its impairment can affect cellular signaling and growth, promoting cancer.

***2.2.2. Enrichment analysis for the identified DEGs between female and male cancer samples***

Based on the observations of each gene in male and female cancer samples, the SR uniquely identified 93 genes that are in the top-500 ranking list. The 93 genes are enriched in cancer pathways such as "Ras signaling pathway (ko04014)", "Renal cell carcinoma (ko05211)", " Non-small cell lung cancer (ko05223)" and " Thyroid cancer (ko05216)". GO enrichment analysis (Fig.S3) highlights biological processes associated with cancer as well. For instance, "Protein ubiquitination" (GO:0016567) and "protein modification by small protein conjugation or removal" (GO:0070647) involve tagging proteins for degradation or modifying their function, which is critical in regulating cell cycle and apoptosis; their dysregulation can lead to cancer. "Cellular catabolic process" (GO:0044248) and "macromolecule catabolic process" (GO:0009057) include the breakdown of cellular components, and their mis-regulation can contribute to tumor growth and metastasis. "Transcription by RNA polymerase III" (GO:0006383) and "ribosome binding" (GO:0043022) are essential for protein synthesis, and abnormalities in these processes can lead to uncontrolled cell proliferation typical of cancer. "Mitochondrial respiratory chain complex III assembly" (GO:0034551) is crucial for cellular energy production, and its dysfunction is often linked to cancer metabolism. "SUMO ligase complex" (GO:0106068) and "Smc5-Smc6 complex" (GO:0030915) are involved in DNA repair and genome stability, and their impairment can cause mutations and cancer progression.

Figure S1. KEGG (A) and GO (B) enrichment analysis for the top-500 ranked 469 genes that are uniquely screened by the SR in cancer and normal samples.

A
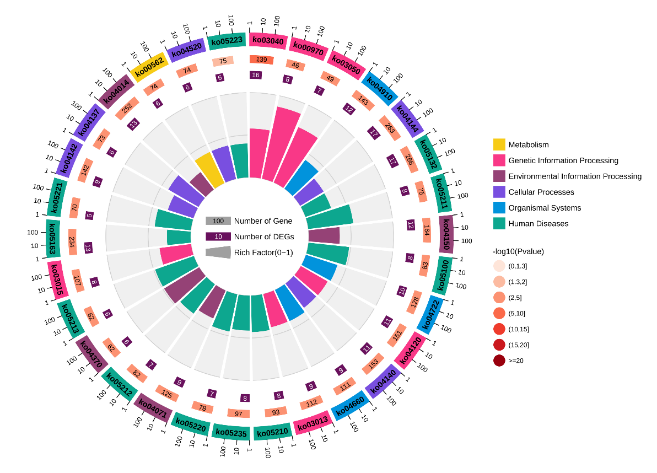
B
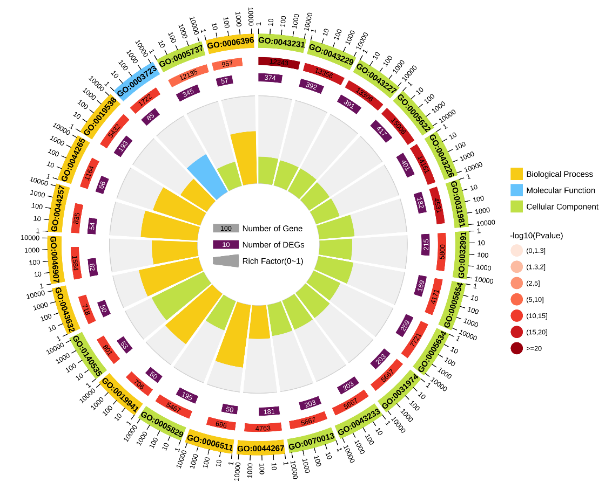


Figure S2. Network diagram for the top-500 ranked 469 genes that are uniquely identified by the SR from the cancer and normal samples. Orange-yellow edges represent the connections that are curated from the database, while purple bolded edges denote pairs of genes that are supported both by the STRING database and the partial KCC. For edges from the STRING database, only gene pairs with the confidence score reaches or exceeds 0.15 are considered, and for the edges that are predicted by the partial KCC, we only consider gene pairs with partial KCC higher than 0.1.


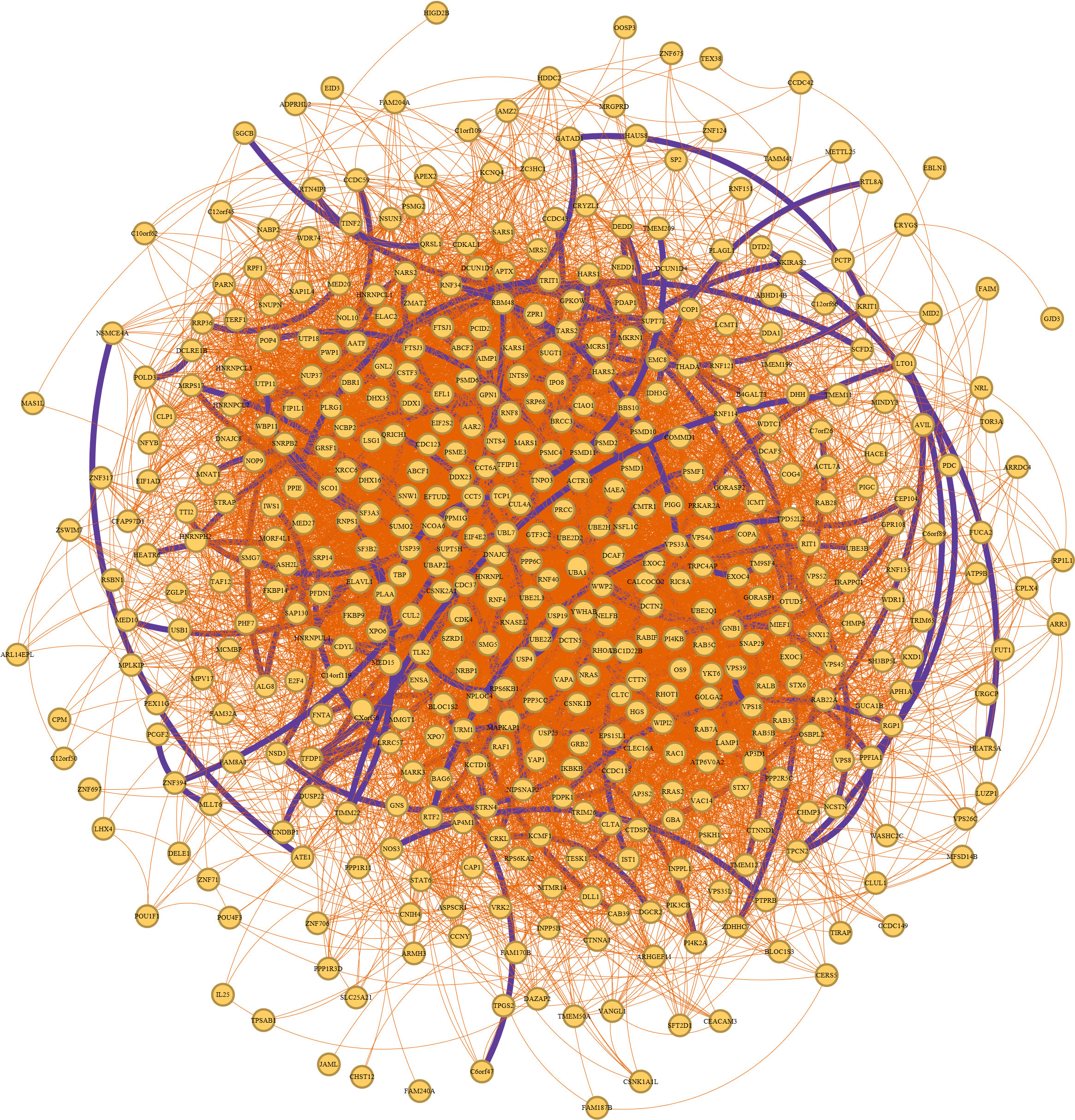


Figure S3. KEGG (A) and GO (B) enrichment analysis for the top ranked 93 genes that are uniquely screened by the SR in female and male cancer samples.

A
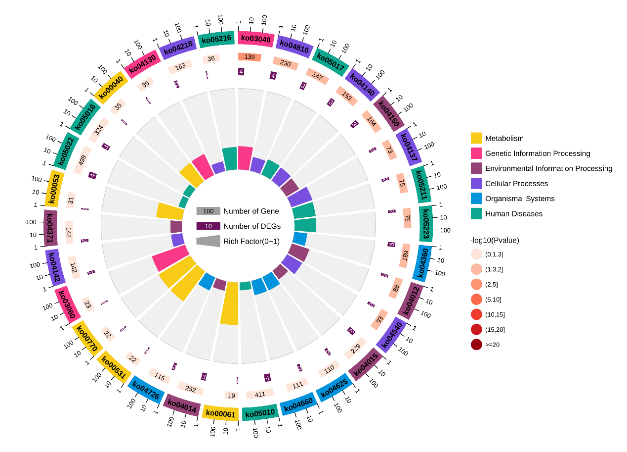
B
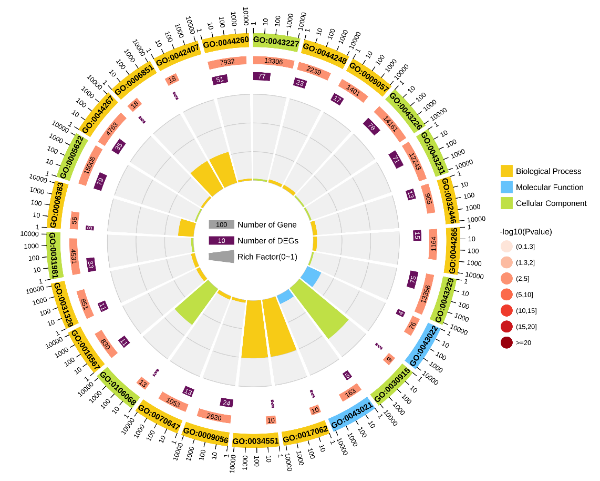


**3. Analysis of the improved** **Naïve Bayes in simulated data**

To demonstrate the effectiveness of the improved Naïve Bayes (INB) model, we conducted simulations using randomly generated two binary-class datasets, each containing 960 samples (480 class 1 and 480 class 2 samples). Each sample comprised two variables, with the following distributions:

**Dataset 1**: Both variables followed normal distributions.

**Dataset 2**: One variable followed a log-normal distribution, and the other followed a generalized extreme value distribution.

***3.1. Performance of the INB on normally distributed simulated data***

For Dataset 1, the probability density function (PDF) $f_{ij}(x)$ for variable *j* in class *i* samples is set as follows:

$$\begin{matrix} f_{11}(x)=\frac{1}{\sqrt{1.0682\pi}}\exp\left(-\frac{\left(x+0.0205\right)^{2}}{1.0682}\right),\text{ }f_{21}(x)=\frac{1}{\sqrt{1.4348\pi}}\exp\left(-\frac{\left(x-2.0338\right)^{2}}{1.4348}\right), \\ f_{12}(x)=\frac{1}{\sqrt{0.9653\pi}}\exp\left(-\frac{\left(x+0.0446\right)^{2}}{0.9653}\right),\text{ }f_{22}(x)=\frac{1}{\sqrt{1.4662\pi}}\exp\left(-\frac{\left(x-1.9879\right)^{2}}{1.4662}\right). \end{matrix}$$

For a class 1 sample$X=(x_{1},x_{2})'=(0.6359,0.1600)'$, assuming we do not know the sample class in advance, substituting these values into the above PDFs, we obtain:

$$\begin{matrix} f_{11}(0.6359)=0.3647,\text{ }f_{21}(0.6359)=0.1207, \\ f_{12}(0.1600)=0.5499,\text{ }f_{22}(0.1600)=0.0477. \end{matrix}$$

Assuming the prior probabilities for the two classes are *q_1_=q_2_=1/2*. Based on Eq. (7) in the main text, we obtain:

$$\begin{matrix} P(1|X)=\frac{q_{1}f_{11}(x_{1})f_{12}(x_{2})}{q_{1}f_{11}(x_{1})f_{12}(x_{2})+q_{2}f_{21}(x_{1})f_{22}(x_{2})}=\frac{0.5\times0.3647\times0.5499}{0.5\times0.3647\times0.5499+0.5\times0.1207\times0.0477}=0.9721, \\ P(2|X)=\frac{q_{2}f_{21}(x_{1})f_{22}(x_{2})}{q_{1}f_{11}(x_{1})f_{12}(x_{2})+q_{2}f_{21}(x_{1})f_{22}(x_{2})}=\frac{0.5\times0.1207\times0.0477}{0.5\times0.3647\times0.5499+0.5\times0.1207\times0.0477}=0.0279. \end{matrix}$$

Obviously, *P(1|X)>P(2|X)*. The sample X should be classified as a class 1 sample.

***3.2. Performance of the INB on non-normally distributed simulated data***

For Dataset 2, the PDF $f_{ij}(x)$ for variable *j* in class *i* samples is set as follows (Variable 1 follows a generalized extreme value distribution, and variable 2 follows a log-normal distribution):

$$\begin{matrix} f_{11}(x)=\frac{1}{0.9393}\times\left(1-0.5375\times\frac{x+0.0473}{0.9393}\right)^{-1-1/0.5375}\exp\left(-\left(1-0.5375\times\frac{x+0.0473}{0.9393}\right)^{-1/0.5375}\right), \\ f_{21}(x)=\frac{1}{0.9743}\times\left(1+0.1486\times\frac{x-0.5130}{0.9743}\right)^{-1+1/0.1486}\times\exp\left(-\left(1+0.1486\times\frac{x-0.5130}{0.9743}\right)^{1/0.1486}\right), \\ f_{12}(x)=\frac{1}{1.0737\times x\sqrt{2\pi}}\exp\left(-\frac{\left(\ln x-0.8605\right)^{2}}{2\times1.0737}\right), \\ f_{22}(x)=\frac{1}{0.8874\times x\sqrt{2\pi}}\exp\left(-\frac{\left(\ln x-1.0992\right)^{2}}{2\times0.8874}\right). \end{matrix}$$

Similar to the above subsection 3.1, for a class 1 sample $X=(x_{1},x_{2})'=(0.6338,0.4348)'$, assuming we do not know the sample class in advance, substituting these values into the above PDFs, we obtain:

$$\begin{matrix} f_{11}(0.6338)=0.3566,\text{ }f_{21}(0.6338)=0.2877, \\ f_{12}(0.4348)=0.2248,\text{ }f_{22}(0.4348)=0.1262. \end{matrix}$$

Assuming the prior probabilities for the two classes are *q_1_=q_2_=1/2*. Based on Eq. (7) in the main text, we obtain:

$$\begin{matrix} P(1|X)=\frac{q_{1}f_{11}(x_{1})f_{12}(x_{2})}{q_{1}f_{11}(x_{1})f_{12}(x_{2})+q_{2}f_{21}(x_{1})f_{22}(x_{2})}=\frac{0.5\times0.3566\times0.2248}{0.5\times0.3566\times0.2248+0.5\times0.2877\times0.1262}=0.6883, \\ P(2|X)=\frac{q_{2}f_{21}(x_{1})f_{22}(x_{2})}{q_{1}f_{11}(x_{1})f_{12}(x_{2})+q_{2}f_{21}(x_{1})f_{22}(x_{2})}=\frac{0.5\times0.2877\times0.1262}{0.5\times0.3566\times0.2248+0.5\times0.2877\times0.1262}=0.3117. \end{matrix}$$

Obviously, *P(1|X)>P(2|X)*. The sample X should be classified as a class 1 sample. If the two variables are incorrectly assumed to follow normal distributions, and after performing distribution fitting, the following fitted PDFs are obtained for the two variables:

$$\begin{matrix} f_{_{11}}^{*}(x)=\frac{1}{\sqrt{4.5603\pi}}\exp\left(-\frac{\left(x-1.4970\right)^{2}}{4.5603}\right),f_{_{21}}^{*}(x)=\frac{1}{\sqrt{1.6112\pi}}\exp\left(-\frac{\left(x-1.2415\right)^{2}}{1.6112}\right), \\ f_{_{12}}^{*}(x)=\frac{1}{\sqrt{1.6783\pi}}\exp\left(-\frac{\left(x-1.5627\right)^{2}}{1.6783}\right),f_{_{22}}^{*}(x)=\frac{1}{\sqrt{1.6590\pi}}\exp\left(-\frac{\left(x-1.6400\right)^{2}}{1.6590}\right). \end{matrix}$$

For the sample $X=(x_{1},x_{2})'=(0.6338,0.4348)'$, substituting the values into the above PDFs, yields:

$$\begin{matrix} f_{_{11}}^{*}(0.6338)=0.2244,\text{ }f_{_{21}}^{*}(0.6338)=0.3534, \\ f_{_{12}}^{*}(0.4348)=0.2038,\text{ }f_{_{22}}^{*}(0.4348)=0.1822. \end{matrix}$$

Assuming the prior probabilities for the two classes are *q_1_=q_2_=1/2*. Based on Eq. (7) in the main text, we obtain:

$$\begin{matrix} P^{*}(1|X)=\frac{q_{1}f_{_{11}}^{*}(x_{1})f_{_{12}}^{*}(x_{2})}{q_{1}f_{_{11}}^{*}(x_{1})f_{_{12}}^{*}(x_{2})+q_{2}f_{_{21}}^{*}(x_{1})f_{_{22}}^{*}(x_{2})}=\frac{0.5\times0.2244\times0.2038}{0.5\times0.2244\times0.2038+0.5\times0.3534\times0.1822}=0.4153, \\ P^{*}(2|X)=\frac{q_{2}f_{_{21}}^{*}(x_{1})f_{_{22}}^{*}(x_{2})}{q_{1}f_{_{11}}^{*}(x_{1})f_{_{12}}^{*}(x_{2})+q_{2}f_{_{21}}^{*}(x_{1})f_{_{22}}^{*}(x_{2})}=\frac{0.3534\times0.1822}{0.5\times0.2244\times0.2038+0.5\times0.3534\times0.1822}=0.5847. \end{matrix}$$

Clearly, *P*(1|X) < P*(2|X)*, indicating that sample X was incorrectly classified as belonging to class 2. This specific example highlights the importance of accurate distribution assumptions in classification. When incorrect distributions are assumed, as demonstrated here, classification accuracy can be compromised. This experiment underscores the ability of the INB to handle categorical data with diverse distributions more effectively than traditional NB models that rely on normal distribution assumptions.
